# CO_2_ capturing by self-assembled belt[14]pyridine encapsulated ionic liquid complexes: a DFT study†

**DOI:** 10.1039/d4ra03394a

**Published:** 2024-10-08

**Authors:** Annum Ahsan, Ahmed Lakhani, Muhammad Umair Ashraf, Muhammad Yar, Sehrish Sarfaraz, Khurshid Ayub

**Affiliations:** a Department of Chemistry, COMSATS University Abbottabad Campus KPK 22060 Pakistan khurshid@cuiatd.edu.pk +92-992-383591; b Department of Biomedical and Health Sciences, Calumet College of St. Joseph Whiting Indiana 46394 USA; c Institute for Applied Physics, Department of Physics, University of Science and Technology Beijing Beijing 100083 China; d Department of Chemistry, Cholistan University of Veterinary and Animal Sciences Bahawalpur Punjab 63100 Pakistan

## Abstract

In the current study, CO_2_ capturing ability of encapsulated ionic liquids (ENILs) *i.e.*, tetramethylammonium chloride (TMACl), 1,3-dimethylimidazolium chloride (MIMCl), and methylpyridinium hexafluorophosphate (MPHP) encapsulated in self assembled belt[14]pyridine (BP) has been studied. The results show that strong van der Waals forces are involved in capturing of CO_2_ by these encapsulated ionic liquids. Strong attractive forces arise from synergistic effect of ionic liquid (encapsulated) and atoms of belt. The interaction energies (*E*_int_) ranging from −12.54 to −18.64 kcal mol^−1^ reveal the capturing of CO_2_ by these systems as thermodynamically feasible process. The type and strength of interactions between CO_2_ and encapsulated ionic liquids is studied through QTAIM and NCI analyses. NCI analysis clearly shows that capturing of CO_2_ is assisted by van der Waals forces between CO_2_ and encapsulated ionic liquid complexes. The same feature is confirmed through QTAIM analysis as well. Natural bond orbital (NBO) analysis' results show the charge transfer between the fragments (encapsulated ionic liquids and CO_2_) which is validated further through electron density differences (EDD) analysis. Overall, transfer of charge towards CO_2_ from encapsulated ionic liquids is proved through the charge accumulation over CO_2_ (*i.e.*, blue isosurfaces on CO_2_ molecules) through EDD analysis. The FMO analyses show the decrease in H–L gaps of encapsulated ionic liquids after CO_2_ capturing. The successful charge transfer and reduction in H–L gap indicate better interaction in the designed systems thus revealing these systems as a potential candidates for CO_2_ capturing. Overall, the best results for CO_2_ capture *i.e.*, the highest interaction energy, the lowest H–L gap, and the strongest forces of interactions are shown by methylpyridinium hexafluorophosphate (MPHP) encapsulated belt[14]pyridine (BP–MPHP) system. This is due to the larger anion of methylpyridinium hexafluorophosphate as compared to the other two encapsulated ionic liquids with Cl^−^ as anion which enables it to develop strong interactions with CO_2_. The designed belt[14]pyridine based encapsulated ionic liquid systems are promising prospects with better CO_2_ capture performance and represent a new entrant in the CO_2_ capturing systems.

## Abstract 2

CO_2_ capturing ability of encapsulated ionic liquids *i.e.*, tetramethylammonium chloride, 1,3-dimethylimidazolium chloride, and methylpyridinium hexafluorophosphate encapsulated in self assembled belt[14]pyridine (BP).

## Graphical abstract

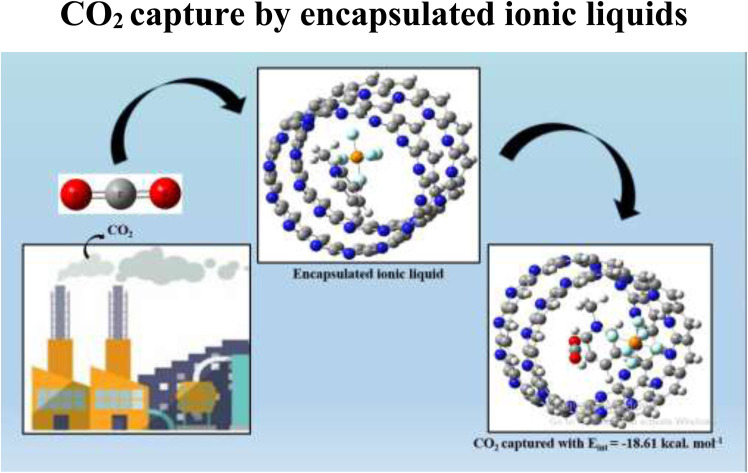

## 1. Introduction

Climate change and global warming issues have now become one of the worldwide topics of interest. The main factor contributing to global warming is increase in concentration of greenhouse gases in atmosphere. The major emission of these gases arises from human activities like automotive vehicles, industrial activities and deforestation *etc.* CO_2_ is one of the major greenhouse gases responsible for global warming^1^ and the methods for Carbon Capture and Storage (CCS) have been under consideration from the last few decades. According to the report presented by National Oceanic and Atmospheric Administration (in November, 2022), the concentration of CO_2_ has reached to 416 ppm in atmosphere. The concentration of CO_2_ is noticeably higher than it was during the pre-industrial revolution era (280 ppm).^2^ CO_2_ is not only responsible for global warming but also considered as an acute toxin that reduces human cognitive ability^3^ and can result in critical physiological symptoms.^4–6^ In order to solve this problem, efforts have been concentrated on exploration of new ways along with improvement in currently used methods for better and more effective capturing and separation of CO_2_. Some of the methods used for capturing of carbon and its further utilization technologies include adsorption,^6,7^ absorption,^8^ membrane separation,^9^ calcium looping,^10^ oxyfuel combustion^11^ and conversion of CO_2_ into fuels and chemicals^12,13^*etc.* Nevertheless, the search for yet better methods and technologies for CO_2_ capture are in progress. Out of all the methods used for CO_2_ capture, absorption by aqueous alkanolamines is considered as benchmark for the industrial processes.^8^ But the limitations associated with this method are corrosivity,^14^ degradation of alkanolamines^15–17^ when used under high temperature and high solvent losses. For the purpose of overcoming these challenges associated with the usage of amines, ionic liquids (ILs) (known as green solvents) have received considerable attention and are considered as the most suitable candidates for CO_2_ capture.

One of the advantages of use of ILs for CO_2_ capturing is their lower energy of regeneration because of the physical absorption mechanism involved. Due to physical absorption, CO_2_ sorption enthalpy for ILs (about 10 to 20 kJ mol^−1^) is much lower than energy required by the standard amine solutions.^18^ Additional benefits of using ILs are, their high thermal stability,^19^ high chemical stability,^20^ recyclability,^20^ non-volatility,^20^ non-flammability and their tunable physicochemical properties.^20^ All these characteristics award ionic liquids great potential for use as absorbents for capturing of CO_2_. Both experimentally and theoretically, CO_2_ capturing by ionic liquids has been studied in detail. One of the reported studies include the interaction between CO_2_ and di-cationic ionic liquids (DILs) showing the effect of cation's symmetry and the length of side chains on interactions between ILs and CO_2_. It is concluded that the symmetric cation with longer side chains tend to interact more strongly with CO_2_ molecules.^21^ Another theoretical study reports the ionic liquids (ILs) and deep eutectic solvents (DESs) as good sources to capture gases. In their study, the environmental friendly and cost-effective cholinium geranate ([Cho][Ger]) IL and cholinium geranate : geranic acid ([Cho][Ger] : Ger acid) DES are investigated for carbon dioxide (CO_2_) capture. The same study concludes that the interaction of CO_2_ is stronger with IL than DES thus introducing a renewable and green IL as an interesting candidate for CO_2_ capture.^22^

Another theoretical study on the mechanism of CO_2_ absorption is reported where dual functional ionic liquids with the combinations of diethylenetriamine cation ([DETAH]^+^) or 1-ethanolamine-ethylenediamine cation ([1-AOEt-EDAH]^+^) and 4-fluorobenzoate anion ([4-F-PhO]) are used.^23^ This study provides detailed explanation on the absorption mechanism of CO_2_ by these ILs. Yet another study involves comparison of CO_2_ absorption by 1,2,4-triazolium-based and imidazolium-based ionic liquids of various anions, namely tetrafluoroborate, bis(trifluoromethylsulfonyl)imide and glycinate. The results reveal that the triazolium-based ionic liquids show higher CO_2_ solubility as compared to imidazolium cation based ionic liquids of different anions.

Despite extensive use of ionic liquids for CO_2_ adsorption, certain disadvantages also exist, including their high viscosity.^24^ The high viscosity of ILs causes low mass transfer rate and may reduce the rate of absorption of CO_2_. Moreover, the high viscosity causes corrosion of equipment, the maintenance costs to be higher,^25–28^ and also increase power consumption. In this regard, a related concept *i.e.*, encapsulation of ionic liquids (ENILs) is considered as a feasible substitute in order to overcome the rate limitation of mass transfer for separation processes that depend upon ionic liquids.^29^ Encapsulated ionic liquids have large surface area and are quite easy to handle.^30^ Moreover, they have improved energy storage, solubility of gases, and extraction capability as compared to un-encapsulated ionic liquids.^30^ They contain solid support having ionic liquids incorporated in the form of micro drops.^31^ They are advanced materials that can be applied in well-established technologies.^32^ The applications of encapsulated ionic liquids include their use in sewage purification,^33^ in gas separation^34^ and as catalysts.^35^ They are also used for capturing of CO_2_. For example, for the first time, Shirato and Satoh^36^ prepared encapsulated microcapsules by the blending of 1-butyl-3-methylimidazolium bis[(trifluoromethyl)sulfonyl]imide with hydrophobic silica nanoparticles at a very high speed. While, Romanos prepared nanoparticles of silica encapsulating ammonium ionic liquids.^37^ According to Romanas' study, ENILs with 40% ILs loading show potential to separate CO_2_ from N_2_. It was concluded that encapsulated ionic liquids show higher absorption capacity for CO_2_*i.e.*, 1.5–3.3 mmol g^−1^ as compared to the conventional ILs.^37^ Ionic liquids encapsulated in assembled belt[14]pyridine (BP) have been investigated recently but have not been used for capturing of gases. The specialty of belt molecules is the fully conjugated π-system with outstanding complexation properties. They play a vital role in supramolecular chemistry.^38^ The defined space/cavity inside the belt molecules awards them capability of forming complexes with various molecules that fit the size of the cavity. Moreover, the space/cavity inside the belts can be deepened through stacking or assembly of belts. Keeping in view the literature studies that encapsulated ILs provide better results for capturing of gases, we have used the newly designed encapsulated ILs *i.e.*, assembled belt[14]pyridine (BP) encapsulated ionic liquids (BP–ILs) for CO_2_ capture in the current study. Our work involves detailed study on interaction of CO_2_ with BP–ILs.

## 2. Methodology

Geometry optimization of all the BP–IL–CO_2_ complexes (encapsulated ionic liquids with CO_2_ captured) has been performed by using Gaussian 09.^39^ DFT method *i.e.*, Becke's three parameter hybrid with Lee–Yang–Parr correlation (B3LYP) along with Pople split valence basis set 6-311G(d,p) are used. The previous studies reveal this level of theory as the most suitable one for ionic liquids^23,40^ because a good agreement to the experimental data was found.^41–43^ Nanobelts have also been studied theoretically frequently at this level of theory as per the literature review.^44,45^ However, keeping in mind the noteworthy dispersion forces present in the designed structures, we've calculated the interaction energies by taking dispersion correction into account. For this purpose, we used Grimme's DFT-D3 method and finally optimized the structures at B3LYP-D3/6-311 G(d,p) level of theory. For the purpose of visualization of structures, generation of input files and for analyzing the output files, software GaussView 5.0 is used.^46^ The presence of true minima and stable optimized structures was ensured by the absence of imaginary frequencies in the calculated vibrational frequencies. The interaction energies (*E*_int_) can be used to understand the strength of interaction between encapsulated ionic liquids (BP–ILs) and CO_2_ which is defined as the difference between the total energy of the complex (BP–IL–CO_2_) and sum of the energies of encapsulated ionic liquids (BP–IL) and CO_2_ molecule. The formula used for Δ*E*_int_ calculation is,

1 Δ*E*_int_ = *E*_complex_ − (*E*_BP–IL_ + *E*_CO_2__)

Quantum chemical calculations performed on the complexes containing fragments in the structure (interacting with each other) are more susceptible towards basis set superposition error (BSSE). Hence, such cases demand corrections. In this regard, counter poise method has been considered as the appropriate method to correct the energy. The following equation is used in this method,

2 Δ*E*_int,CP_ = *E*_int_ − *E*_BSSE_

For detailed investigation of transfer of charge between the fragments (BP–ILs and CO_2_), the analysis of natural bond orbitals (NBOs) is carried out. Moreover, for the purpose of further investigation and visualization of the interactions between fragments in terms of the accumulation and depletion of charge, the electron density differences (EDD) analysis is employed. Furthermore, for examination of changes in electronic properties after BP–ILs and CO_2_ interact with each other, frontier molecular orbitals (FMOs) analysis is performed.^47,48^

In the capturing of CO_2_, major role is played by noncovalent interactions between BP–ILs and CO_2_. Therefore, it is important to estimate the interactions involved in CO_2_ capturing. For this purpose, the non-covalent interaction (NCI) analysis is used which distinguishes and visualizes different nonbonding interaction forces *i.e.*, repulsive forces, van der Waals forces, and electrostatic interactions. Through NCI analysis, we get 2-D reduced density gradient (RDG) plots along with 3-D isosurfaces of BP–ILs–CO_2_ which are generated with the help of Multiwfn 3.8 software.^49^ The 2-D RDG graphs depend on electron density (*ρ*) and reduced density gradient.^50^ Their mathematical relationship is given as:3
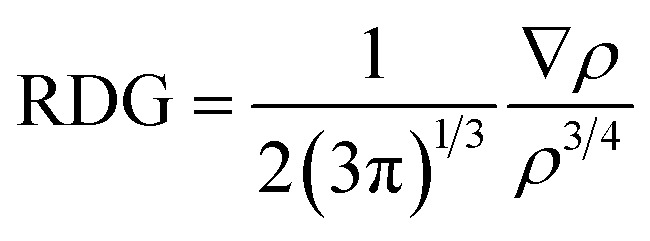


The noncovalent interactions' nature is evaluated by the help of color scheme, which depends on the value of sign(*λ*_2_)*ρ*. In 3-D NCI plots, green, blue, and red isosurfaces are associated with small negative value, higher negative value, and higher positive value of sign(*λ*_2_)*ρ* in RDG plots, respectively. The green color indicates weak van der Waals interactions, blue color indicates strong electrostatic interactions, and red isosurfaces show repulsive forces.

For further exploration of noncovalent interactions' nature between CO_2_ and BP–ILs, quantum theory of atoms in molecules (QTAIM) analysis is used. In QTAIM, different topological parameters *i.e.*, electron density (*ρ*), Laplacian of electron density (∇^2^*ρ*), kinetic energy density (Lagrangian) *G*_(r)_, potential energy density *V*_(r)_, and total energy density *H*_(r)_ are calculated in order to understand nature of interactions through bond critical points (BCPs).^51,52^

## 3. Results and discussion

### 3.1 Geometric properties

**Fig. 1:**
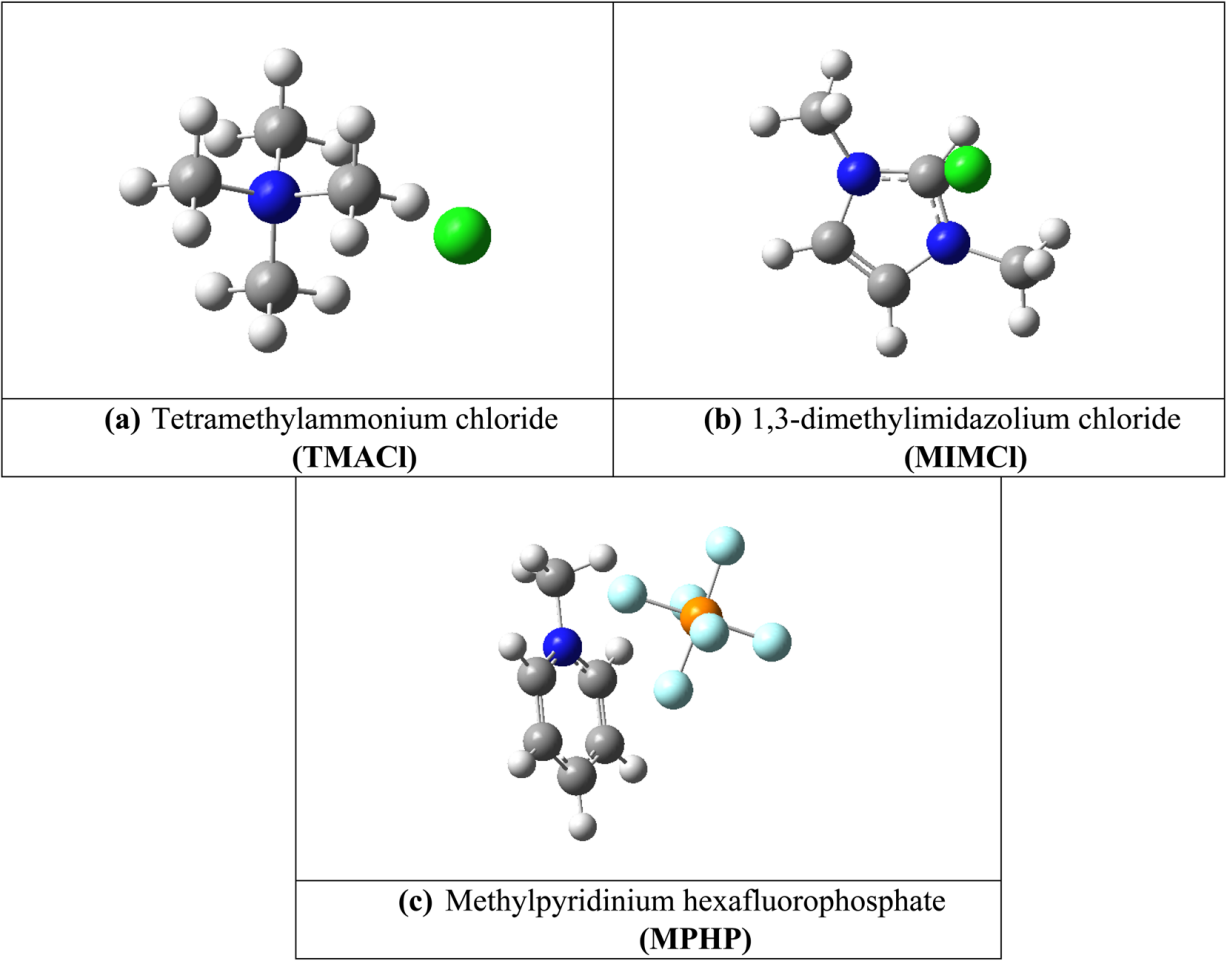
The optimized structures of ILs; (a) tetramethylammonium chloride (TMACl), (b) 1,3-dimethylimidazolium chloride (MIMCl) and (c) methylpyridinium hexafluorophosphate (MPHP).

**Fig. 2:**
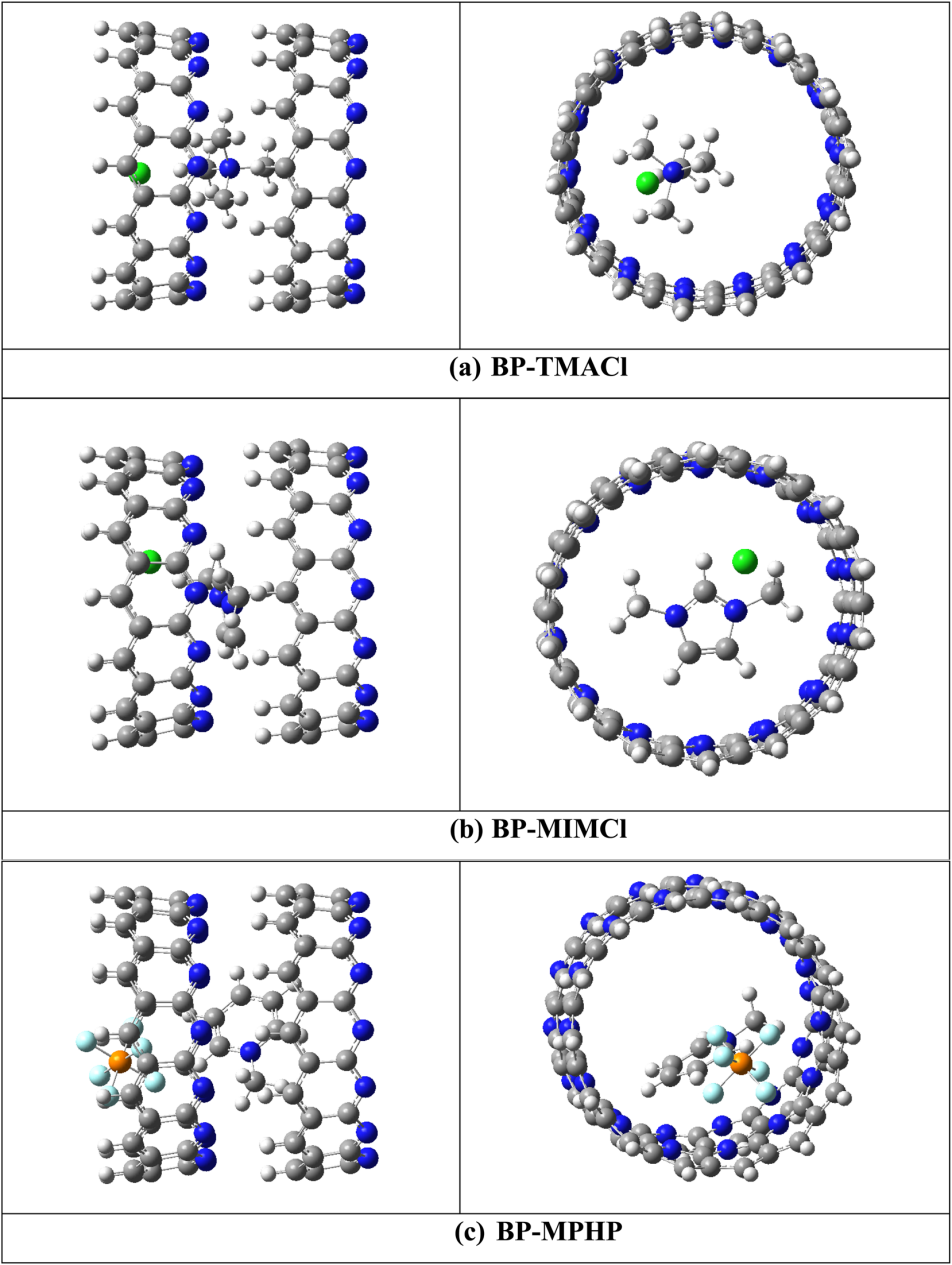
Optimized structures of BP–ILs where IL = TMACl (a), MIMCl (b) and MPHP (c).

Assembled nanobelt[14]pyridine encapsulated ionic liquids (ENILs) are used for carbon dioxide (CO_2_) capture in the current study. These ENILs are, tetramethylammonium chloride (TMACl) encapsulated belt[14]pyridine (BP–TMACl), 1,3-dimethylimidazolium chloride (MIMCl) encapsulated belt[14]pyridine (BP–MIMCl), and methylpyridinium hexafluorophosphate (MPHP) encapsulated belt[14]pyridine (BP–MPHP). The mentioned encapsulated ionic liquids have been designed by our group recently. Fig. 2 shows the encapsulated ionic liquids while Fig. 1 presents the geometries of non-encapsulated ionic liquids.

**Fig. 3:**
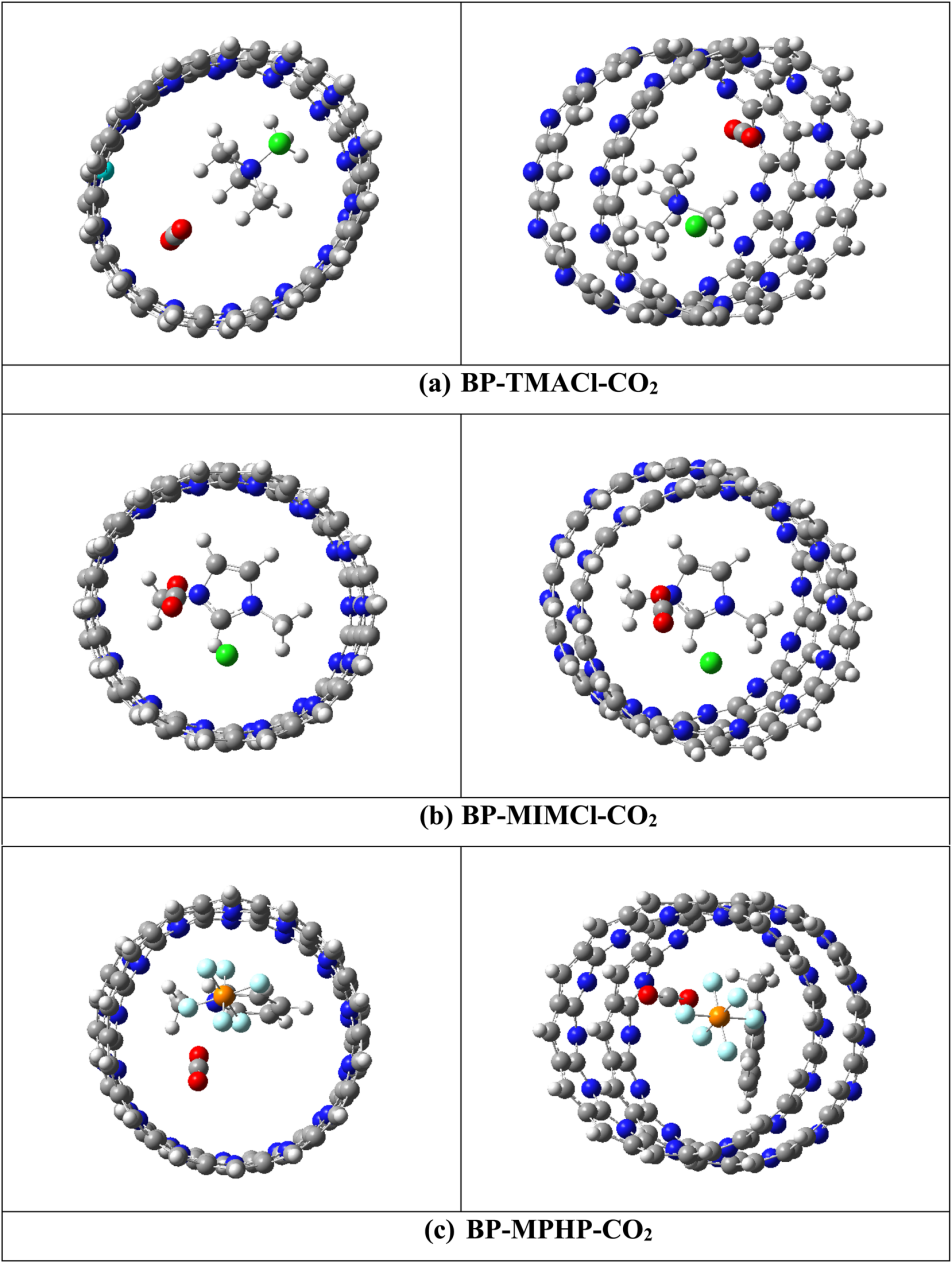
Optimized structures of BP–IL–CO_2_ complexes.

For capturing of CO_2_ by these encapsulated ionic liquids, two different initial orientations have been selected for CO_2_ with respect to the encapsulated ionic liquids (Fig S1†). The first orientation contains CO_2_ near cation of ionic liquid while second orientation contains CO_2_ near anion of ionic liquid inside the belts' cavity. In case of tetramethylammonium chloride encapsulated belt[14]pyridine, the results show that the orientation containing CO_2_ near cation is more stable one and interaction is more exothermic for CO_2_ capturing in this orientation. The results in case of 1,3-dimethylimidazolium chloride and methylpyridinium hexafluorophosphate encapsulated belt[14]pyridine show that better capturing properties along with more exothermic interactions are displayed when CO_2_ is kept near anions (oriented more towards anion and oriented slightly away from cation). The cation with concentrated charge (tetramethylammonium) attracts CO_2_ more strongly as compared to anion as in case of tetramethylammonium chloride encapsulated belt[14]pyridine. The reason can be the two electronegative electron rich oxygen atoms of CO_2_ which show more attraction towards the cation (with concentrated charge) as compared to anion). While, in case of 1,3-dimethylimidazolium chloride encapsulated belt[14]pyridine and methylpyridinium hexafluorophosphate encapsulated belt[14]pyridine, the cations are 1,3-dimethylimidazolium and methylpyridinium which contain a ring structure with charge delocalized over it. The delocalization affects the intensity of the charge of cation *i.e.*, lowers its impact and reduces the interaction between CO_2_ and cation. Hence, anions in these cases show stronger interaction with CO_2_. The optimized structures with the stable orientations for CO_2_ capturing are presented in Fig. 3.

Overall, the process of capturing CO_2_ by all the three encapsulated ionic liquids is thermodynamically feasible as revealed through negative interaction energies ranging from −12.54 to −18.61 kcal mol^−1^ (calculated using eqn (1)). The negative interaction energies point toward the exothermic nature and experimental feasibility of these reactions. The trend of interaction energies followed by BP–ILs–CO_2_ complexes is BP–MPHP–CO_2_ (−18.61 kcal mol^−1^) > BP–MIMCl–CO_2_ (−14.43 kcal mol^−1^) > BP–TMACl–CO_2_ (−12.64 kcal mol^−1^). CO_2_ captured by methylpyridinium hexafluorophosphate encapsulated belt[14]pyridine shows largest interaction energy due to the larger anion of methylpyridinium hexafluorophosphate as compared to the other two encapsulated ionic liquids with Cl^−^ as anion. Larger anion of methylpyridinium hexafluorophosphate enables it to develop strong interaction with CO_2_, both cation and anion play their role in capturing of CO_2_ hence awarding better CO_2_ capturing properties to methylpyridinium hexafluorophosphate encapsulated belt[14]pyridine.

As the designed complexes contain fragments, hence basis set superposition error (BSSE) corrected interaction energies have been calculated (Table 1). The BSSE corrected interaction energies involved in CO_2_ capturing process range from −10.36 to −12.63 kcal mol^−1^. Comparing BSSE energies with uncorrected energies (*E*_int_), we see that the absolute values of BSSE energies are altered slightly.

**Table 1:** Interaction energy, *E*_int(BP–IL–CO_2_)_ (in kcal mol^−1^), basis set superposition error corrected interaction energies, BSSE (kcal mol^−1^), OCO bend angle, energy of HOMO, *E*_H_ (in eV), energy of LUMO, *E*_L_ (in eV) and HOMO–LUMO gap, H–L gap (in eV) of BP–ILs–CO_2_ systems

BP–IL–CO_2_	*E*_int (BP–IL–CO2)_	BSSE	OCO bend angle	*E*_H_	*E*_L_	H–L gap
CO_2_	—	—	—	−10.25	0.80	9.45
BP–TMACL	—	—	—	−5.39	−5.12	0.27
BP–TMACL–CO_2_	−12.64	−10.36	174	−5.40	−5.14	0.26
BP–MIMCL	—	—	—	−5.39	−5.12	0.27
BP–MIMCL–CO_2_	−14.43	−12.63	173	−5.40	−5.14	0.26
BP–MPHP	—	—	—	−5.41	−5.12	0.29
BP–MPHP–CO_2_	−18.61	−11.22	178	−5.39	−5.14	0.25

Moreover, the adsorbed CO_2_ also shows bending after interacting with the encapsulated ionic liquids. The angle of CO_2_ is changed from 180° to 173°, 174° and 178° for BP–MIMCl–CO_2_, BP–TMACl–CO_2_ BP–MPHP–CO_2_, respectively. The change in angle shows better interaction of CO_2_ with encapsulated ionic liquid systems.

### 3.2 Electronic properties

#### 3.2.1. Natural bond orbital analysis (NBO)

The detailed NBO analysis shows change in NBO charges of ionic liquids in BP–IL–CO_2_ complexes (encapsulated ionic liquids with CO_2_ captured) as compared to charges on encapsulated ionic liquids before CO_2_ capturing (BP–ILs) thus showing the charge transfer between ionic liquids and captured CO_2_. Moreover, change in magnitude of NBO charges on the atoms of belt in BP–ILs–CO_2_ complexes (encapsulated ionic liquids with CO_2_ captured) as compared to encapsulated ionic liquids (without CO_2_) is also observed. This shows that the captured CO_2_ interacts with both ionic liquids and the belt atoms.

In case of CO_2_ capturing by tetramethylammonium chloride encapsulated belt[14]pyridine (BP–TMACl), the belt atoms in BP–TMACl show the negative charge ranging from −0.417 to −0.422|*e*| on all the nitrogen atoms that are pointing outward (outer nitrogen) whereas the nitrogen atoms that lie at the joining points of two nanobelt units (inner nitrogen) bear negative charges of −0.510 to −0.525|*e*|. After introducing CO_2_, the NBO charges on belt atoms decrease slightly. The negative charge ranges from −0.417 to −0.421|*e*| and −0.509 to −0.525|*e*| on outer and inner nitrogen atoms, respectively in BP–TMACl–CO_2_ complex. Discussing the interaction between ionic liquid and CO_2_, the charge on O atoms of CO_2_ has increased from −0.499|*e*| to −0.529|*e*| and −0.508|*e*| while the negative charge on the chloride (Cl^−^) of ionic liquid tetramethylammonium chloride has decreased from −0.941|*e*| to −0.934|*e*|. Moreover, the positive charge on carbon atom of CO_2_ has increased from 0.998|*e*| (uncaptured carbon) to 1.028|*e*| (in captured carbon). This shift of charge from carbon can be seen on the carbon atoms of the belt surrounding captured CO_2_ molecule. CO_2_ molecule has not only interacted with ionic liquid but its interaction with the belt atoms in terms of charge transfer can also be observed. Overall, the charge analysis shows that CO_2_ has accumulated the charge from tetramethylammonium chloride encapsulated belt[14]pyridine.

After introducing CO_2_ to 1,3-dimethylimidazolium chloride encapsulated belt[14]pyridine (BP–MIMCl), the nitrogen atoms show slight increase in negative charge from −0.41–−0.416|*e*| to −0.414–−0.417|*e*| on outer nitrogen atoms of the assembled belts. Discussing the interaction between 1,3-dimethylimidazolium chloride and CO_2_, the charge on O atoms of CO_2_ has increased from −0.499|*e*| to −0.516|*e*| and −0.524|*e*| while the negative charge on the Cl^−^ of ionic liquid (1,3-dimethylimidazolium chloride) has decreased from −0.943|*e*| to −0.942|*e*|. Moreover, the positive charge on carbon atom of CO_2_ has increased from 0.998|*e*| (uncaptured carbon) to 1.033|*e*| (in captured carbon). Overall, the charge analysis shows that CO_2_ molecule has accumulated the charge from 1,3-dimethylimidazolium chloride encapsulated belt[14]pyridine.

In case of BP–MPHP–CO_2_ complex (methylpyridinium hexafluorophosphate encapsulated belt[14]pyridine with captured CO_2_), the outer nitrogen atoms of belts show an increase in magnitude of negative charge from −0.413 to −0.419|*e*| (for BP–MPHP) to the range of −0.416 to −0.419|*e*| while inner nitrogen atoms show an increase in charge up to −0.533 to −0.512|*e*| from −0.503 to −0.534|*e*| (for BP–MPHP) after capturing CO_2_. Discussing the interaction between methylpyridinium hexafluorophosphate and CO_2_, the charge on O atoms of CO_2_ has increased from −0.499|*e*| to −0.524|*e*| and −0.505|*e*| while the negative charge on the F-atoms of anion of ionic liquid has decreased. Moreover, the positive charge on carbon atom of CO_2_ has increased from 0.998|*e*| (uncaptured carbon) to 1.026|*e*| (in captured carbon). In this case, again CO_2_ accumulates the charge from methylpyridinium hexafluorophosphate encapsulated belt[14]pyridine.

The NBO analysis shows that CO_2_ molecule has not just interacted with ionic liquid but its interaction with the atoms of belt is also observed through charge transfer between them. This is why, in the current study, the interaction energies of encapsulated ionic liquids (BP–ILs) with captured CO_2_ are quite higher than the systems where pure or un-encapsulated forms of ILs are used for CO_2_ capturing.^23,40^

Additionally, there is overall slight change observed in NBO charges over the atoms of complex, although strong interactions have been detected between the fragments. The reason is transfer of charge taking place in both the directions *i.e.*, from CO_2_ towards the atoms of belt & ionic liquid and *vice versa*. When certain amount of charge is transferred from CO_2_ molecule towards the atoms of encapsulated ionic liquids and at the same time, charge is transferred backwards as well, then overall change in NBO charge becomes lower. Hence, we can say that exchange of charges is taking place which leads to the net effect of slight change in charge. Moreover, despite overall slight change in NBO charges, CO_2_ molecule shows more charge accumulation as compared to the depletion of charge.

#### 3.2.2. Electron density differences analysis

**Fig. 4:**
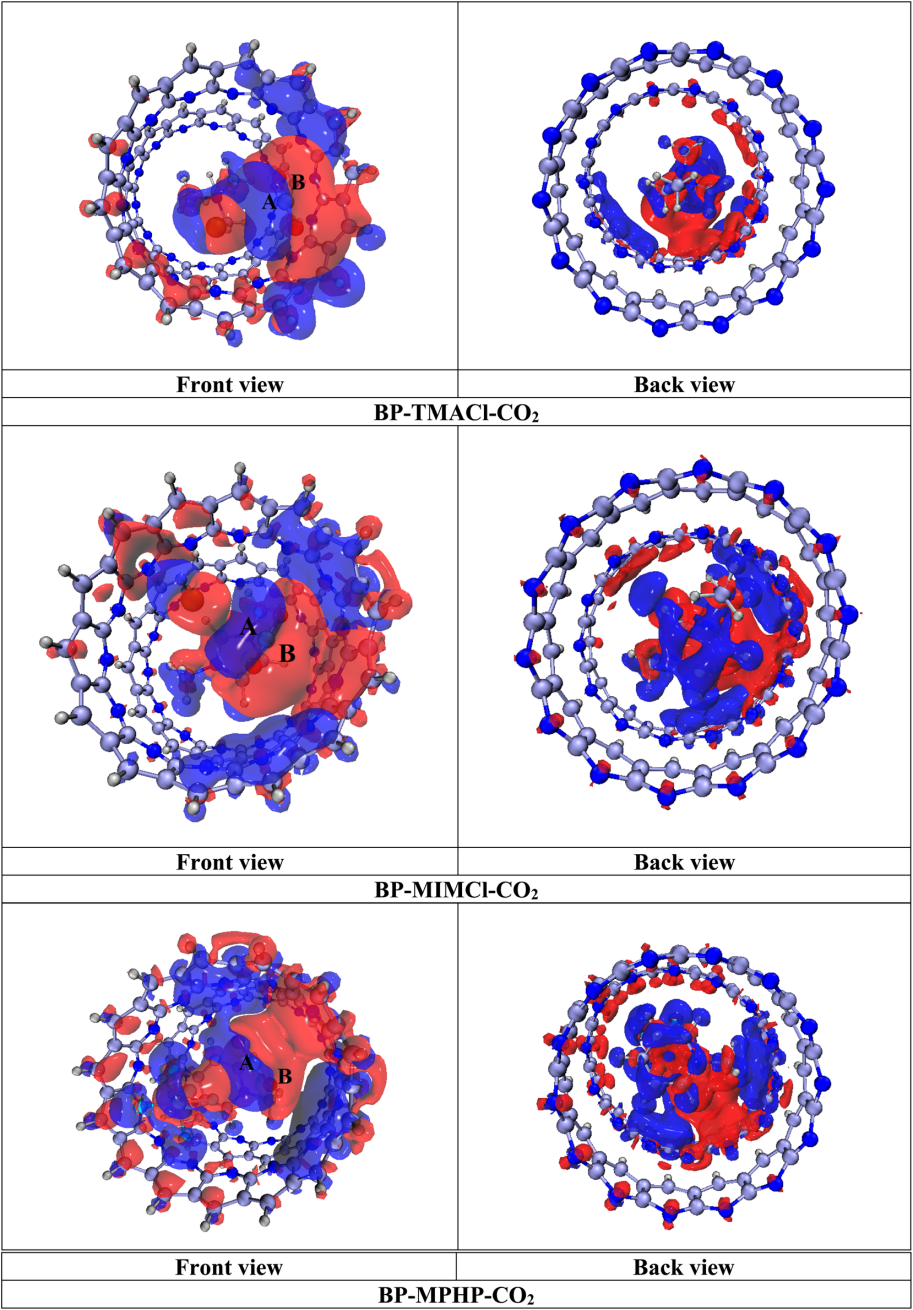
EDD isosurfaces of BP–ILs–CO_2_ complexes: red color shows the electron density depletion, whereas blue isosurfaces indicate the accumulation of electron density, (isovalue = 0.00006 a.u.).

The purpose of EDD analysis is to acquire the visual illustration of charge transfer between encapsulated ionic liquids (BP–ILs) and CO_2_. EDD analysis gives us the isosurfaces containing two colors *i.e.*, red and blue. The red isosurfaces display depletion of electronic density while, the blue isosurfaces show increase in electron density or the accumulation of charge. In the designed systems, we can see both the isosurfaces *i.e.*, red and blue. This indicates that exchange of charges has taken place between the components of system *i.e.*, encapsulated ionic liquids and CO_2_. The same feature *i.e.*, the exchange of charges between components is also validated through NBO analysis. As EDD analysis shows the charge transfer in both ways (exchange of charges between fragments) hence, from the generated figures, we can see that CO_2_ molecule has accumulated both the red and blue isosurfaces over it in all the three complexes. This shows that it has transferred charge in both the directions *i.e.*, accumulated as well as depleted the charge (as per NBO analysis). In Fig. 4 front view of complexes shows the isosurfaces of both the colors over CO_2_ labelled as A and B. The same results can be observed for the belt and ionic liquids which are surrounded by the isosurfaces of both the colors. Moreover, according to NBO analysis, CO_2_ molecule shows more charge accumulation as compared to the depletion of charge, the results of EDD analysis also show more density of blue colored isosurfaces over CO_2_ as compared to red color. The EDD isosurfaces for BP–ILs–CO_2_ complexes (encapsulated ionic liquid with CO_2_ captured) are given in Fig. 4.

#### 3.2.3. HOMO and LUMO analysis

The interaction between encapsulated ionic liquids (BP–ILs) and captured CO_2_ in terms of frontier molecular orbitals shows that there is no prominent difference in the density or position of HOMO and LUMO of BP–ILs–CO_2_ (assembled belt[14]pyridine complexes with CO_2_ captured) as compared to BP–ILs (encapsulated ionic liquids without captured CO_2_). The capturing of CO_2_ by encapsulated ionic liquids is not causing merging or interaction of the energy levels or orbitals of encapsulated ionic liquids and CO_2_. This is due to greater difference between the energies of HOMO and LUMO of components (encapsulated ionic liquids and CO_2_). Moreover, after CO_2_ capturing by encapsulated ionic liquids, a slight decrease in HOMO–LUMO gap (H–L gap) is observed for BP–IL–CO_2_ systems as compared to encapsulated ionic liquids (without CO_2_) (Table 1).

**Fig. 5:**
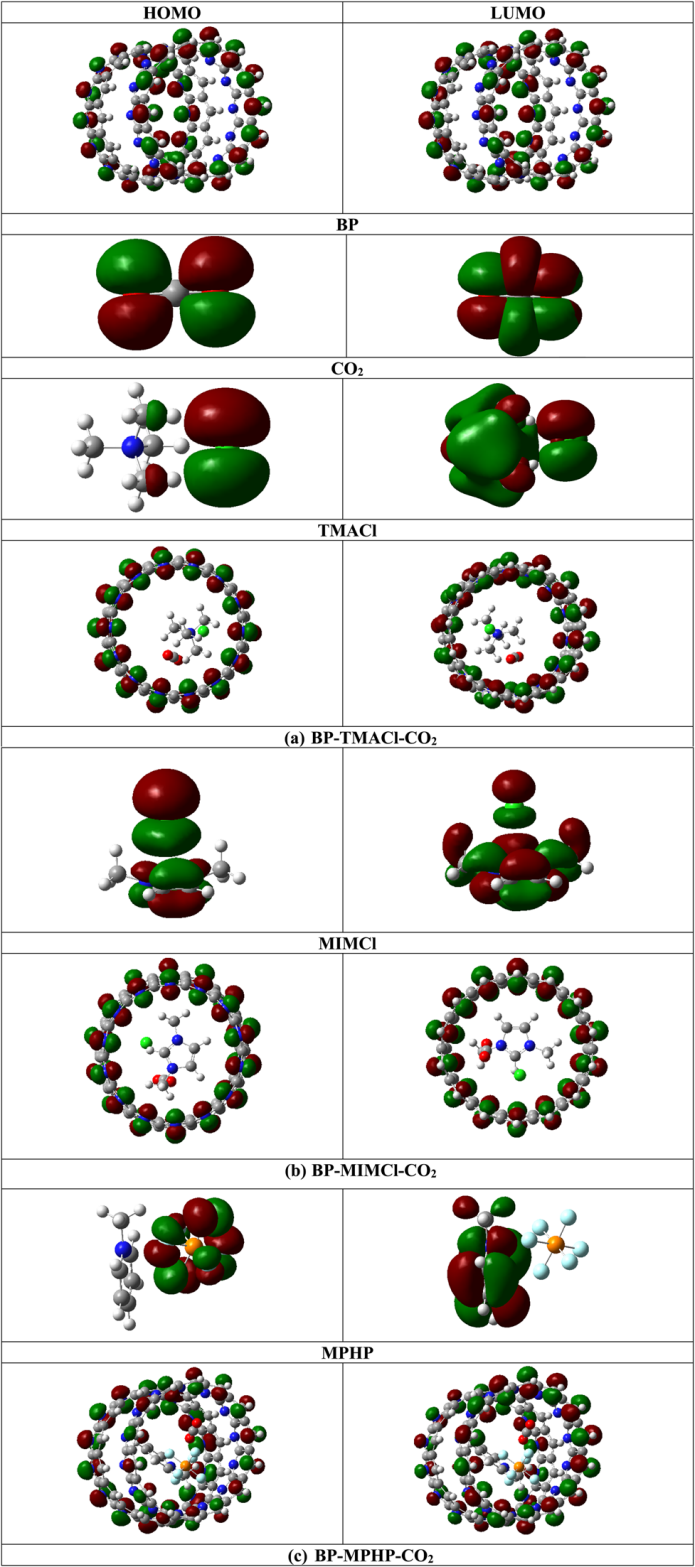
HOMOs and LUMOs of BP–IL–CO_2_ complexes (where IL = TMACl, MIMCl and MPHP).

The H–L gap of encapsulated ionic liquids decreases slightly after CO_2_ capturing. For BP–TMACl–CO_2_, H–L gap decreases to 0.26 eV from 0.27 eV (for tetramethylammonium chloride encapsulated belt[14]pyridine). For BP–MIMCl–CO_2_ and BP–MPHP–CO_2_, the H–L gaps decrease to 0.26 eV and 0.25 eV from 0.27 eV (for 1,3-dimethylimidazolium chloride encapsulated belt[14]pyridine) and 0.29 eV (for methylpyridinium hexafluorophosphate encapsulated belt[14]pyridine), respectively. All the three systems show very slight change in H–L gap. The comparatively greater change in H–L gap is shown by methylpyridinium hexafluorophosphate encapsulated belt[14]pyridine (BP–MPHP) after capturing of CO_2_, showing better interaction of CO_2_ with BP–MPHP as compared to the other two encapsulated ionic liquids. In case of methylpyridinium hexafluorophosphate encapsulated belt[14]pyridine, the energy of HOMO (*E*_H_) is equal to −5.41 eV while its LUMO energy (*E*_L_) is equal to −5.12 eV. After CO_2_ capturing, the *E*_H_ increases to −5.39 eV and *E*_L_ decreases to −5.14 eV. This leads to overall decrease in energy gap. On the other hand, 1,3-dimethylimidazolium chloride encapsulated belt[14]pyridine doesn't show any change in *E*_L_ before and after CO_2_ capturing but shows an increase in *E*_H_ from −5.39 eV (for BP–MIMCl) to −5.38 eV. The increase in *E*_H_ after CO_2_ capture results in overall reduction in H–L gap. For BP–TMACl–CO_2_ complex (tetramethylammonium chloride encapsulated belt[14]pyridine with CO_2_ captured), the *E*_H_ shows decrease in value from −5.39 eV (tetramethylammonium chloride encapsulated belt[14]pyridine) to −5.40 eV. On the other hand, the decrease in *E*_L_ from −5.12 eV to −5.14 eV results in reduction in H–L gap. The values of *E*_H_, *E*_L_ and H–L gaps for uncaptured CO_2_, encapsulated ionic liquids (BP–ILs) and encapsulated ionic liquid with CO_2_ captured (BP–ILs–CO_2_) complexes are given in Table 1 and the densities of HOMO and LUMO are shown in Fig. 5.

### 3.3 Reduced density gradient analysis

**Fig. 6:**
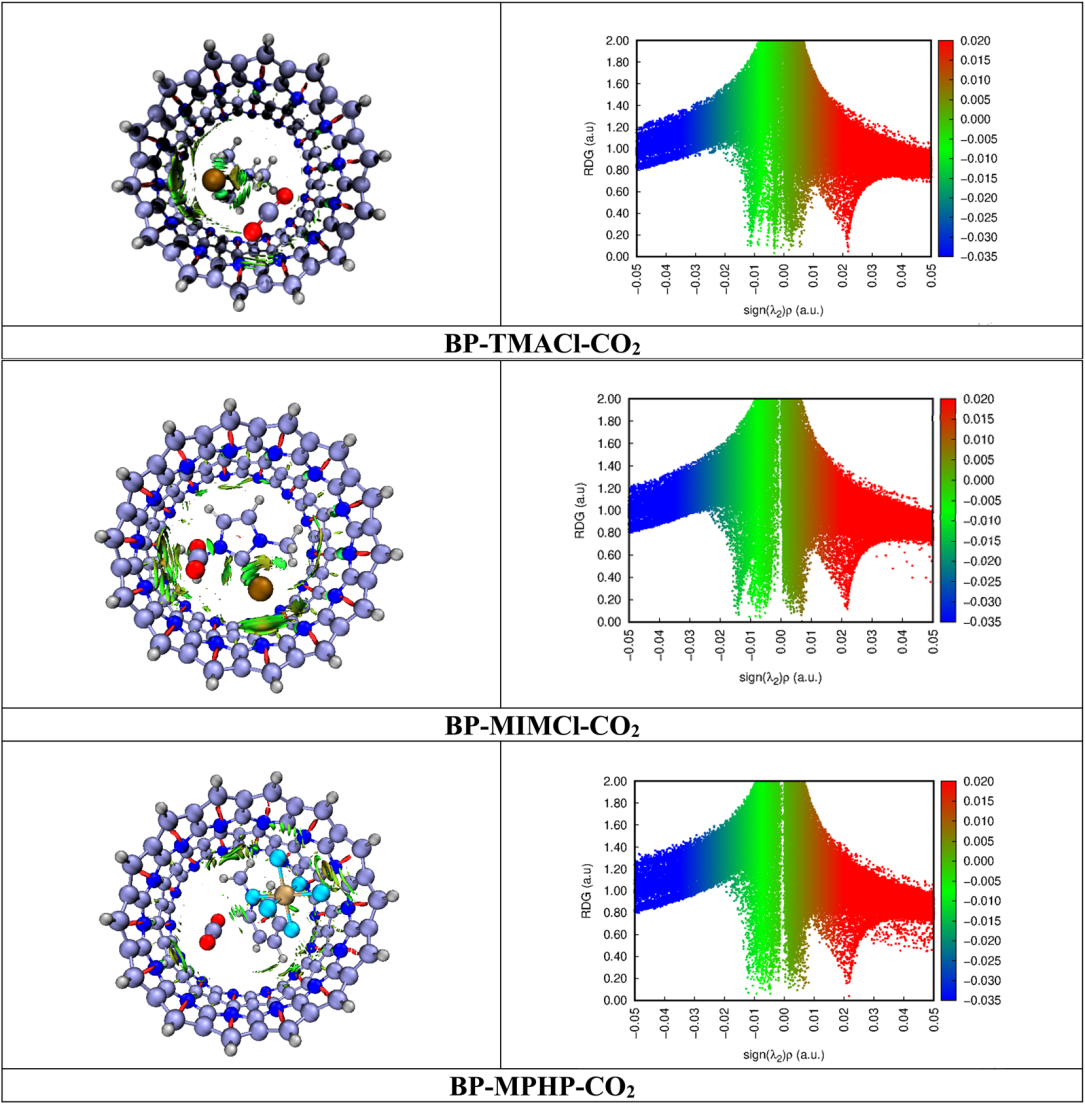
NCI analysis of BP–ILs–CO_2_ complexes with 3-D topological forms and 2-D RDG graphs at an isovalue of 0.5 a.u. where blue color depicts hydrogen bonding, green color indicates van der Waal's forces, and red color is for repulsive forces.

Noncovalent interaction (NCI) analysis is also known as reduced density gradient (RDG) analysis. It is basically a visual approach NCI analysis used for studying the nonbonding interactions in complexes. The analysis evaluates the nature of nonbonding interaction forces in complexes *i.e.*, repulsive forces, van der Waals interactions and hydrogen bonding. Fig. 6 presents the scatter 2-D plots and 3-D topological graphs of studied BP–ILs–CO_2_ complexes (assembled belt[14]pyridine encapsulated ionic liquids with CO_2_ captured). The generated 2-D RDG scattered spectra present green, red, and blue colors as a function of *λ*_2_(*ρ*) in the range of −0.05 a.u. to 0.05 a.u. Blue colored spikes are not observed in these spectra showing the absence of strong electrostatic interactions or hydrogen bonding involved in CO_2_ capturing. The green colored spikes can be seen in the range of 0.01 a.u. to −0.02 a.u. of *λ*_2_(*ρ*). They depict London dispersion forces in the system. Moreover, the red colored spikes present in the spectra at the region of above 0.02 a.u. of *λ*_2_(*ρ*) depict the steric repulsive forces present in the complexes. Fig. 6 shows the 3-D topologies where the light brown and green patches are present. Light brown and green patches are observed in the complex around CO_2_ molecule. The patches are present between CO_2_ and ionic liquids indicating van der Waals interactions between them. These patches are also present between CO_2_ and BP showing CO_2_'s interaction with BP through van der Waals forces. Moreover, in 2-D RDG plots of all the three CO_2_ captured encapsulated ionic liquid (BP–ILs) complexes, the green spikes appear at up to −0.02 a.u., which is indication of strong van der Waals forces having main role in CO_2_ capturing. NCI analysis clearly shows that capturing of CO_2_ is assisted by van der Waals forces between CO_2_ and both ionic liquid and belt[14]pyridine atoms.

### 3.4 QTAIM analysis

For the detailed evaluation of noncovalent interactions further, Bader's QTAIM analysis is considered. The nature of nonbonding interactions is estimated on the basis of bond critical points with the help of a number of topological parameters *i.e.*, electron density (*ρ*), Laplacian ∇^2^(*ρ*), and total electron density *H*_(r)_ which is the sum of local kinetic energy *G*_(r)_ and local potential energy *V*_(r)_.

4 *H*_(r)_ = *V*_(r)_ + *G*_(r)_

The values of these parameters decide the type and strength of interaction forces in complexes. When the values of total energy density *H*_(r)_ and Laplacian ∇^2^*ρ* are positive, the interactions between the fragments forming complexes are noncovalent in nature, whereas negative values of these parameters reveal the presence of covalent bonding. Moreover, total energy density *H*_(r)_ less than zero (*H*_(r)_ < 0) and greater than zero (*H*_(r)_ > 0) reveal shared shell and closed-shell interactions, respectively. Furthermore, the strength of noncovalent interactions is revealed through electron density (*ρ*) *i.e.*, for strong forces of attraction (covalent interactions), the value of electron density has to be positive always (*ρ* > 0.1 a.u.) while for weak forces of attraction (noncovalent interactions), the value of electron density has to be negative always (*ρ* < 0.1 a.u.). Additionally, interaction energy (*E*_int_) of individual bonds also helps in evaluation of nature of bonding (computed *via* Espinosa approach).

5 *E*_int_ (a.u.) = ½*V*_(r)_

The values of *E*_int_ ranging from 3 to 10 kcal mol^−1^ show existence of hydrogen bonding (strong electrostatic interactions). Similarly, another parameter *i.e.*, the ratio −*V*/*G* also helps in evaluation of the nature of interactions, the values of −*V*/*G* < 1 and −*V*/*G* > 2 show nonbonding and covalent interactions, respectively.

**Fig. 7:**
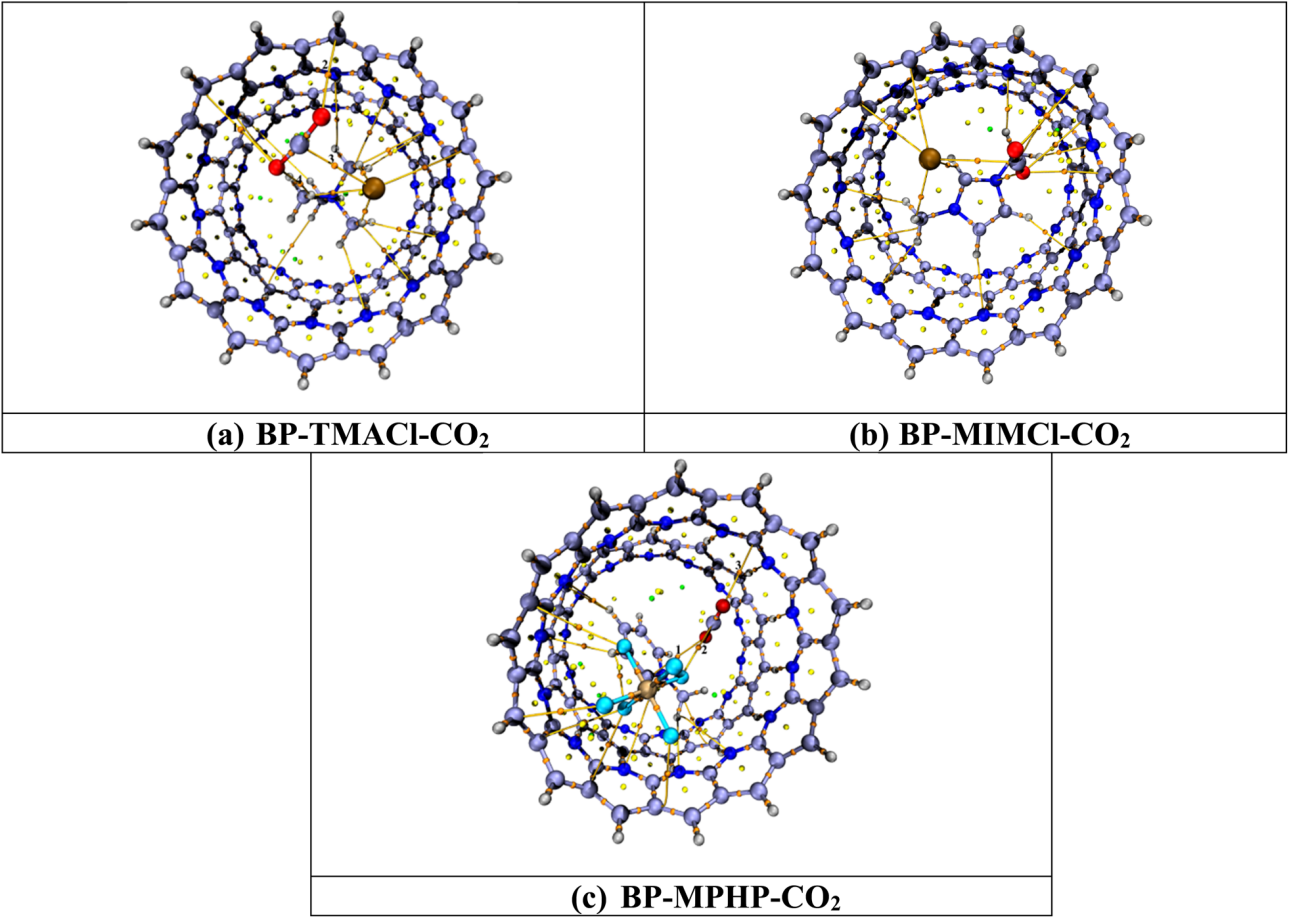
QTAIM analysis results of BP–ILs–CO_2_ complexes. Bond paths are shown by lines between CO_2_ and BP–IL, whereas bond critical points (BCPs) are presented by colored dots.

Fig. 7 shows the topologies calculated with the help of QTAIM analysis while Table 2 contains the BCP parameters. The QTAIM analysis of the BP–ILs–CO_2_ complexes *i.e.*, BP–TMACl–CO_2_, BP–MIMCl–CO_2_ and BP–MPHP–CO_2_ shows that total numbers of BCPs found for BP–TMACl–CO_2_ are four (4), for BP–MIMCl–CO_2_ are five (5) and for BP–MPHP–CO_2_ are three (3). The BCPs are related to the possible number of nonbonding interactions between the CO_2_ molecule and encapsulated ionic liquids, BP–ILs.

**Table 2:** Nonbonding interactions study of BP–ILs–CO_2_ complexes *via* QTAIM analysis

BP–ILs–CO_2_	BP–ILs–CO_2_	*ρ* (a.u.)	∇^2^*ρ* (a.u.)	*G*_(r)_ (a.u.)	*V*_(r)_ (a.u.)	*H*_(r)_ (a.u.)	−*V*/*G*	*E*_int_ (kcal mol^−1^)
BP–TMACl–CO_2_	C159⋯C158	0.009	0.031	0.006	−0.005	0.0016	−3.125	−1.569
	O160⋯C90	0.004	0.012	0.002	−0.002	0.0006	−3.333	−0.628
	O161⋯C112	0.002	0.008	0.002	−0.001	0.0004	−2.500	−0.314
	O161⋯H146	0.003	0.011	0.002	−0.002	0.0005	−4.000	−0.628
BP–MPHP–CO_2_	C163⋯F161	0.009	0.044	0.009	−0.008	0.0016	−0.889	−2.510
	O164⋯C141	0.008	0.029	0.006	−0.005	0.0012	−0.833	−1.569
	O165⋯C88	0.007	0.026	0.006	−0.005	0.0009	−0.833	−1.569
BP–MIMCl–CO_2_	C158⋯Cl155	0.010	0.034	0.007	−0.005	0.0016	−0.71	−1.569
	O159⋯N147	0.007	0.027	0.006	−0.005	0.0009	−0.83	−1.569
	O159⋯C7	0.005	0.019	0.004	−0.003	0.0008	−0.75	−0.941
	O159⋯C82	0.006	0.020	0.004	−0.003	0.0008	−0.75	−0.941
	O160⋯C87	0.006	0.019	0.004	−0.003	0.0008	−0.75	−0.941

The topological parameters presented in Table 2 show that the CO_2_ capturing by assembled belt[14]pyridine encapsulated ionic liquids is assisted by strong van der Waals interaction forces. For all the three systems designed for CO_2_ capturing *i.e.*, tetramethylammonium chloride encapsulated belt[14]pyridine, 1,3-dimethylimidazolium chloride encapsulated belt[14]pyridine, and methylpyridinium hexafluorophosphate encapsulated belt[14]pyridine, both total energy density *H*_(r)_ and Laplacian ∇^2^*ρ* are positive showing noncovalent interactions involved in CO_2_ capturing. The electron density values for BP–ILs–CO_2_ complexes (encapsulated ionic liquid complexes with CO_2_ captured) are less than 0.1 a.u. but not negative *i.e.*, ranging from 0.002–0.010 a.u. These values for electron density show presence of strong noncovalent interactions involved in CO_2_ capturing and among three systems, the values are overall higher for CO_2_ captured methylpyridinium hexafluorophosphate encapsulated belt[14]pyridine (BP–MPHP–CO_2_) revealing the strongest interaction in this system as compared to the other two. Moreover, the calculated *E*_int_ (values *via* Espinosa approach) for our designed systems range from −0.314 to −2.510 kcal mol^−1^ indicating presence of van der Waals forces. These values are greatest *i.e.*, ranging from −1.569–2.510 kcal mol^−1^ in case of BP–MPHP–CO_2_ (similar to the *E*_int_ calculated through eqn (1)). In the same way, the values of other topological parameters for BP–MPHP–CO_2_ are also higher as compared to CO_2_ captured 1,3-dimethylimidazolium chloride encapsulated belt[14]pyridine complex (BP–MIMCl–CO_2_) and CO_2_ captured tetramethylammonium chloride encapsulated belt[14]pyridine complex (BP–TMACl–CO_2_). Hence, the strongest van der Waals forces of attraction are present in BP–MPHP–CO_2_. Likewise, the higher values calculated for Laplacian ∇^2^(*ρ*) and electron density (*ρ*) also point toward the stronger interaction between CO_2_ and methylpyridinium hexafluorophosphate encapsulated belt[14]pyridine. We conclude through QTAIM analysis that stronger van der Waals forces are playing vital role in CO_2_ capturing in our designed systems and methylpyridinium hexafluorophosphate encapsulated belt[14]pyridine is the best choice for CO_2_ capturing. The results computed with the help of QTAIM analysis can be strongly correlated to the results of interaction energies and NCI analysis.

## 4. Conclusions

In the current study, three different ILs encapsulated in self-assembled belt[14]pyridine have been studied for CO_2_ capturing. The encapsulated ionic liquids are, tetramethylammonium chloride (TMACl), 1,3-dimethylimidazolium chloride (MIMCl) and methylpyridinium hexafluorophosphate (MPHP) encapsulated in self-assembled belt[14]pyridine. The interaction energies ranging from −12.54 to −18.61 kcal mol^−1^ show thermodynamic feasibility of capturing of CO_2_ by these systems. Moreover, the detailed study of CO_2_ capturing involves NBO and FMO analysis. NBO analysis shows the charge transfer between fragments (BP–ILs and CO_2_) which is further confirmed through EDD analysis. FMO analysis reveals slight reduction in H–L gap after capturing of CO_2_ by encapsulated ionic liquids (BP–ILs). With the largest *E*_int_ calculated for BP–MPHP–CO_2_, the maximum reduction in H–L gap is also shown by the same system after CO_2_ capturing *i.e.*, from 0.29 eV to 0.25 eV. Furthermore, the nature and strength of forces involved in CO_2_ capturing process are studied through NCI and QTAIM analysis. The results of both the analyses show that strong van der Waals forces are involved in capturing process in all the systems. The strongest interactions are observed in BP–MPHP–CO_2_ system. Overall, the system showing largest interaction energy (−18.61 kcal mol^−1^), the lowest H–L gap (0.25 eV) and strongest forces of interactions for CO_2_ capturing is methylpyridinium hexafluorophosphate encapsulated self-assembled belt[14]pyridine based system. The study provides satisfactory results for CO_2_ capturing by the novel encapsulated ionic liquids systems.

## Data availability

The data that supports the findings of this study are available from the corresponding author upon reasonable request.

## Conflicts of interest

There are no conflicts to declare.

## Supplementary Material

RA-014-D4RA03394A-s001
