# Supplementary material for: CO2 capturing by self-assembled belt[14]pyridine encapsulated ionic liquid complexes: a DFT study
Source: RSC Adv. 2024 Oct 8;14(43):31837–49. doi: 10.1039/d4ra03394a (PMC11459277; doi:10.1039/d4ra03394a)
Supplement: RA-014-D4RA03394A-s001 [file RA-014-D4RA03394A-s001.pdf]

Supplementary Information (SI) for RSC Advances.
This journal is © The Royal Society of Chemistry 2024

CO_2 capturing by self-assembled belt[14]pyridine encapsulated ionic liquid complexes: A DFT study

Annum Ahsan[a], *Ahmed Lakhani*[b], *Muhammad Umair Ashraf*[c], *Muhammad Yar*[a, d], *Sehrish Sarfaraz*[a] & *Khurshid Ayub*[a]*

[a] Department of Chemistry, COMSATS University, Abbottabad Campus, KPK, Pakistan 22060

[b] Department of Biomedical and Health Sciences, Calumet College of St. Joseph, Whiting, Indiana 46394, United States

[c] Institute for Applied Physics, Department of Physics, University of Science and Technology Beijing, Beijing 100083, China

[d] Department of Chemistry, Cholistan University of Veterinary and Animal Sciences, Bahawalpur, Punjab, Pakistan 63100

Correspondence: khurshid@cuiatd.edu.pk (K. A.)

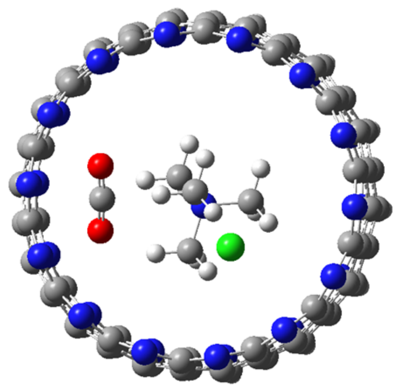

(a) BP-TMACl-CO_2 (CO_2 near cation)

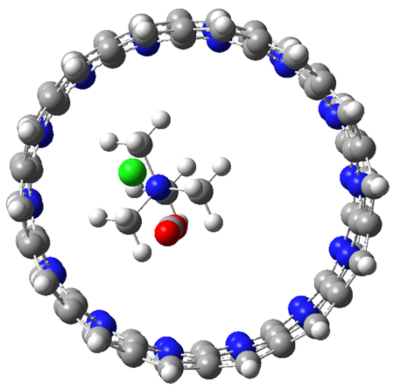

(b) BP-TMACl-CO_2 (CO_2 near cation)

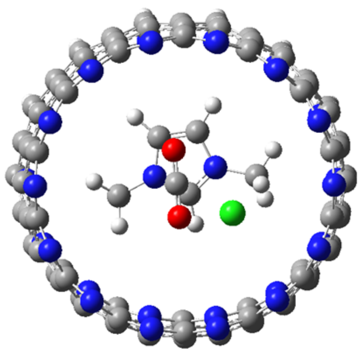

(c) BP-MIMCl-CO_2 (CO_2 near cation)

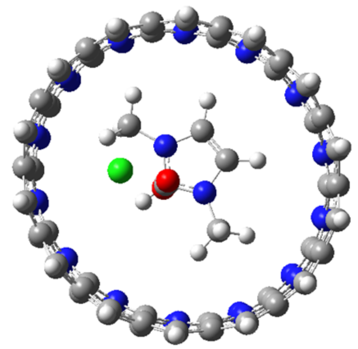

(d) BP-MIMCl-CO_2 (CO_2 near anion)

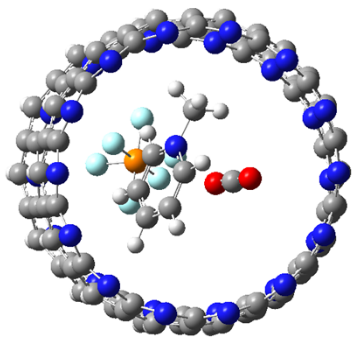

(e) BP-MPHP-CO_2 (CO_2 near cation)

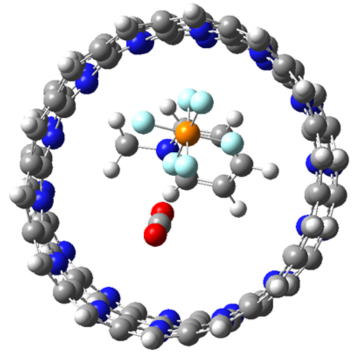

(f) BP-MMPHP-CO_2 (CO_2 near anion)

Fig. S1: Different initial orientations for CO_2 capturing by encapsulated ionic liquids.

AIMD, Ab initio molecular dynamics analysis

We have studied the thermal stability of a belt[14]pyridine-encapsulated ionic liquids after CO_2 capture using ab initio molecular dynamics (AIMD) simulations. The simulations are performed using HSE06 hybrid functional along with projected augmented wave potential (pseudopotential) as implanted in VASP code [1]. HSE06 is a hybrid functional which is considered best to study geometrical, electronic and thermodynamic properties of the systems. HSE06 effectively captures exchange and correlation function which provides balance between local and non-local exchange contributions [2, 3].

Using the Nosé–Hoover heat bath scheme at 300 K with a 1 fs time step over 1 ps, we found that the average temperature and total free energy fluctuations remained nearly constant (Fig. S2 and S3), indicating thermal stability. The final structures (Fig. S2) attained as a result of simulation present minor distortion in the structures. Overall, the results show feasibility of the reaction of capturing of CO_2 by encapsulated ionic liquids.

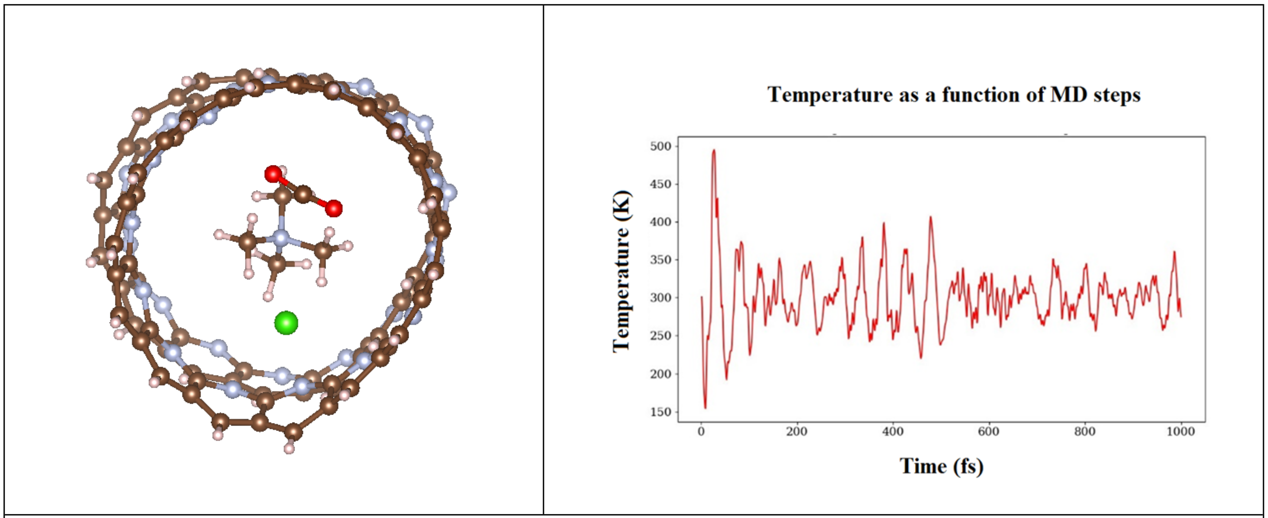

BP-TMACl-CO_2

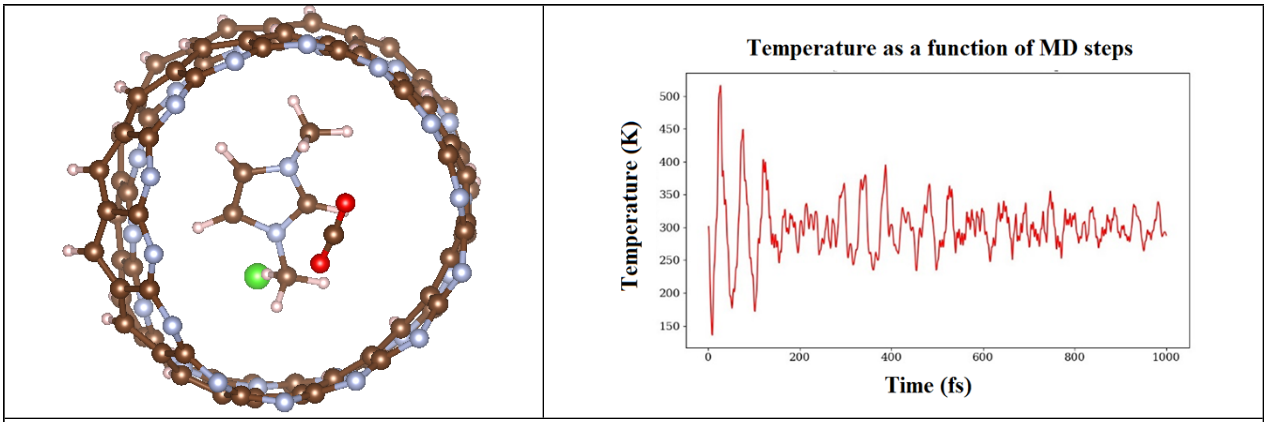

BP-MIMCl-CO_2

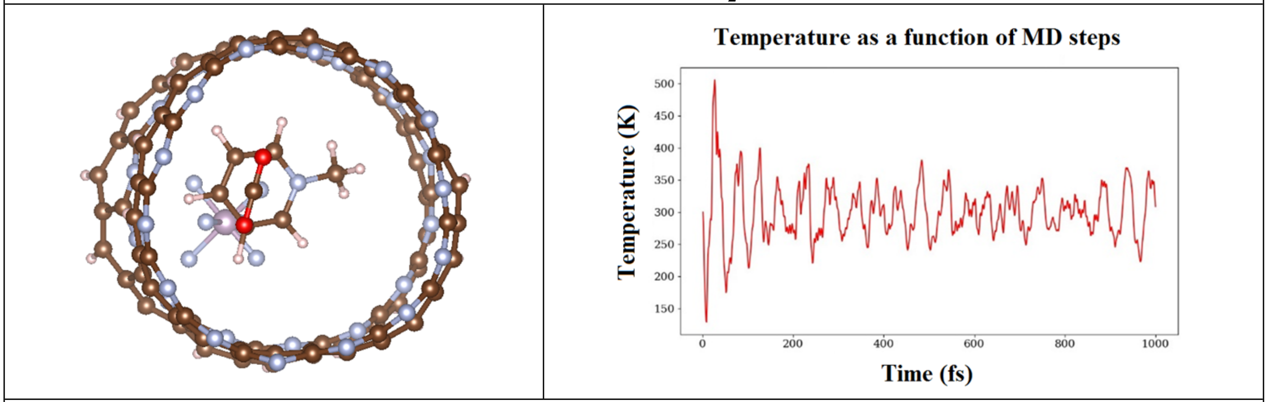

BP-MPHP-CO_2

Fig. S2: Results of AIMD analysis studied at 300 K with a 1 fs time step.

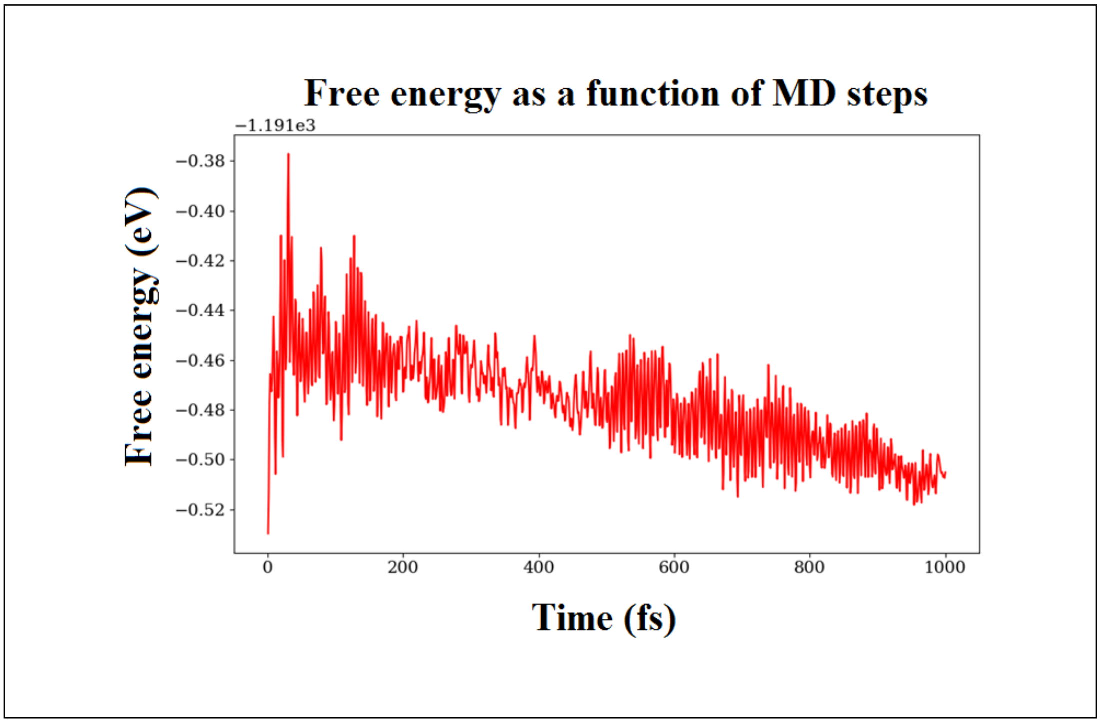

BP-TMACl-CO_2

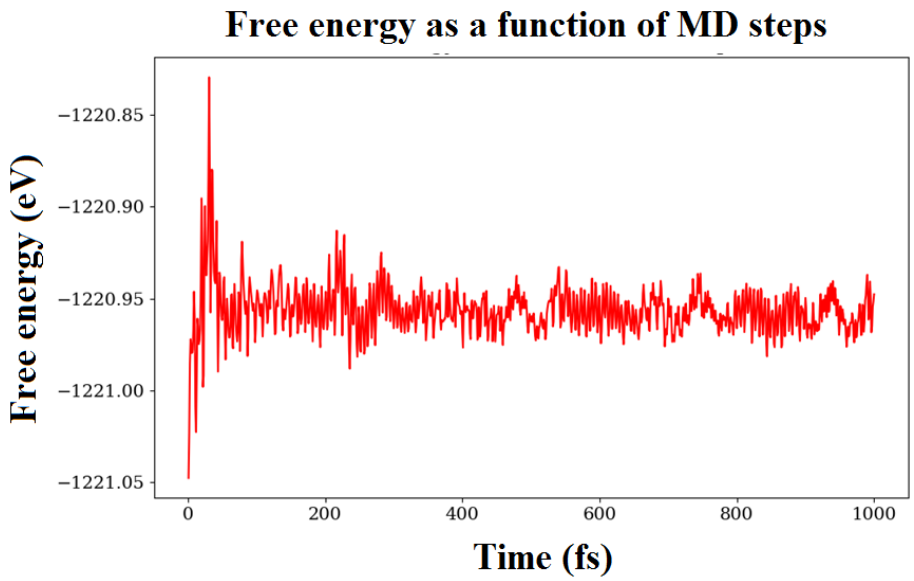

BP-MIMCl-CO_2

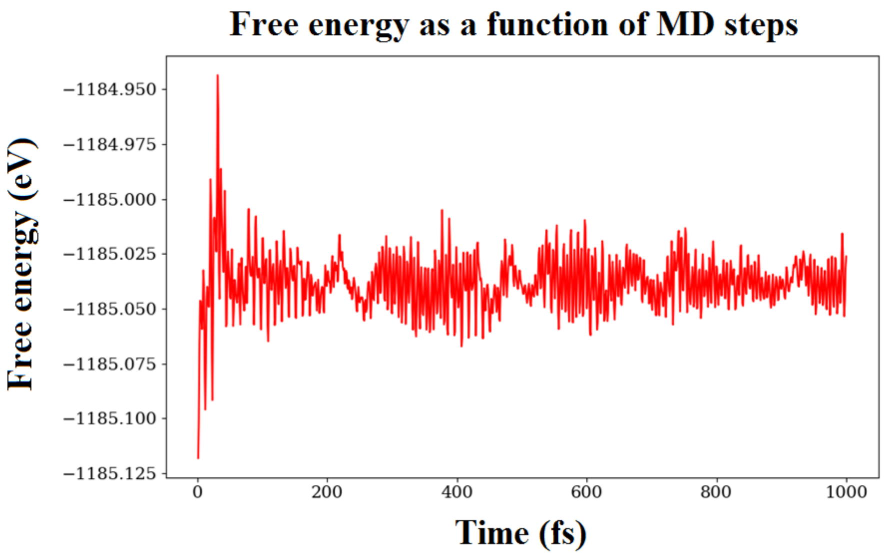

BP-MPHP-CO_2

Fig. S3: Free energy as a function of MD time (in fs) at the temperature of 300K for BP-IL-CO_2 systems.

BP-TMACl-CO_2 (Orientation 1-near cation)

C	1.81769700	-4.56522200	4.09305200
C	2.02507000	-4.72016300	2.64328500
C	3.17954700	-4.18408100	2.06606800
C	3.96795900	-3.33303000	2.84633300
C	3.66645800	-3.20956800	4.28264500
C	4.80416200	-2.38423900	2.24914400
C	5.10494500	-1.23044100	2.97972800
C	4.73277900	-1.17847200	4.40359100
C	5.42131500	-0.03697700	2.32255600
C	5.12357500	1.16175300	2.97880800
C	4.75004300	1.11667500	4.40252000
C	-0.43013000	-5.02409300	3.89786100
C	-0.31730800	-5.18445600	2.43865500
C	0.94834300	-5.12920600	1.85107600
C	-2.67291700	-4.52617600	3.74200700
C	-2.65291200	-4.67299600	2.27736400
C	-1.47566400	-5.09980400	1.66055900
C	-4.51152100	-3.14586800	3.64699400
C	-4.58575700	-3.26081500	2.18143600
C	-3.69290900	-4.11287100	1.52916900
C	4.84095000	2.31944300	2.24708400
C	4.01846600	3.28100500	2.84288500
C	3.71401300	3.16317600	4.27902600
C	-5.56455400	-1.10431000	3.60798400
C	-5.71883400	-1.15288600	2.14565700
C	-5.30812400	-2.30118500	1.46568700
C	3.24303500	4.14251200	2.06109600
C	2.09583600	4.69554000	2.63687500
C	1.88529400	4.54527500	4.08666900
C	1.02547200	5.11837600	1.84333700
C	-0.23968200	5.19215600	2.42999600
C	-0.35556800	5.03617300	3.88944500
C	-1.39860200	5.12195500	1.65135100
C	-2.58212900	4.71306700	2.26828800
C	-2.60514800	4.56994000	3.73326200
C	-3.62949600	4.16603600	1.52073000
C	-4.53514200	3.32871600	2.17438500
C	-4.46363700	3.21631900	3.64028900
C	-5.27141400	2.37832700	1.46049200
C	-5.70009000	1.23811400	2.14293500
C	-5.54711200	1.19060800	3.60544200
C	-5.91186300	0.04340200	1.45009600

H	3.33666000	-4.24466000	0.99195800
H	5.01622600	-2.43099000	1.18318900
H	5.65730900	-0.03956700	1.26059300
H	1.05586700	-5.19102300	0.77139000
H	-1.41183300	-5.15446800	0.57708200
H	-3.67333800	-4.16166300	0.44362100
H	5.05503000	2.36133800	1.18130800
H	-5.33761100	-2.33685300	0.38001200
H	3.40253300	4.19909200	0.98711300
H	1.13545800	5.17683300	0.76372800
H	-1.33245900	5.17340000	0.56790500
H	-3.60766100	4.21210900	0.43515600
H	-5.29927400	2.41239100	0.37474900
H	-5.97102700	0.04297700	0.36537200
N	-1.61775700	-4.85844100	4.50372300
N	2.72152800	-3.95646700	4.87722900
N	4.74698400	-0.03062000	5.10004600
N	2.77964500	3.92437900	4.87218000
N	-1.54580700	4.88871300	4.49491400
N	-5.10957800	2.24733300	4.31008000
N	-5.14261200	-2.16584700	4.31488500
N	-5.66807200	0.04477100	4.29634800
N	-3.62154400	3.96560100	4.37089600
N	0.72269200	4.87543800	4.67341500
N	4.23032300	2.18126700	5.03510200
N	4.19768400	-2.23466800	5.03743800
N	0.65082100	-4.87770300	4.68096300
N	-3.67999600	-3.90565500	4.37886800
C	2.37541400	-4.36635700	-2.05545600
C	2.57903100	-4.56925300	-3.49600300
C	3.76506100	-4.09715000	-4.06685300
C	4.60049200	-3.28047100	-3.30607600
C	4.31738400	-3.13625400	-1.87194900
C	5.50039000	-2.38822900	-3.89674400
C	5.87836700	-1.25814800	-3.17334000
C	5.53896800	-1.20085300	-1.74573500
C	6.21647600	-0.06800600	-3.82155400
C	5.91719900	1.13092700	-3.17098800
C	5.57469900	1.08143100	-1.74387600
C	0.11035700	-4.78853000	-2.24287900
C	0.22054800	-5.02098300	-3.68980600
C	1.49483100	-4.99955600	-4.26376200
C	-2.15310400	-4.36110600	-2.40812300
C	-2.14155200	-4.57910800	-3.86232300
C	-0.95275700	-5.01726500	-4.45103500
C	-4.06792300	-3.08520400	-2.53634400

C	-4.13647600	-3.23935800	-3.99792300
C	-3.22596300	-4.10463900	-4.60968800
C	5.57128900	2.27203500	-3.89347900
C	4.69199200	3.18469700	-3.30325100
C	4.40386800	3.04654700	-1.86956400
C	-5.21324800	-1.09463700	-2.61262000
C	-5.31579100	-1.14767700	-4.07893100
C	-4.89903900	-2.31616000	-4.72373200
C	3.87349900	4.01660400	-4.06561200
C	2.69543700	4.51021200	-3.49644100
C	2.48696100	4.31352500	-2.05572800
C	1.61975300	4.95727100	-4.26615000
C	0.34557200	5.00283200	-3.69320000
C	0.23005400	4.77560900	-2.24592100
C	-0.82679800	5.01966900	-4.45533800
C	-2.02466700	4.60708800	-3.86647700
C	-2.04139300	4.39154400	-2.41203500
C	-3.11888300	4.15559300	-4.61362300
C	-4.04974800	3.31301900	-4.00093100
C	-3.98462000	3.15853300	-2.53929700
C	-4.83551900	2.40873200	-4.72585700
C	-5.28303000	1.25244000	-4.07994900
C	-5.18149300	1.19805400	-2.61362000
C	-5.51101900	0.05491100	-4.76785400
H	3.93547000	-4.20654500	-5.13332600
H	5.70678000	-2.44934900	-4.96035600
H	6.44038700	-0.07059500	-4.88322700
H	1.61126700	-5.14501200	-5.33348000
H	-0.90296100	-5.17348400	-5.52449500
H	-3.23968700	-4.23516200	-5.68761500
H	5.77976900	2.32847900	-4.95691300
H	-4.95157600	-2.38844700	-5.80588800
H	4.04663300	4.12135100	-5.13207900
H	1.73955500	5.09815700	-5.33609300
H	-0.77324100	5.17276200	-5.52906800
H	-3.12944100	4.28515700	-5.69170600
H	-4.88642000	2.48148400	-5.80806600
H	-5.57486300	0.05534600	-5.85178100
N	-1.08005200	-4.61400800	-1.64585700
N	3.31334500	-3.80245700	-1.27995800
N	5.63420000	-0.06158300	-1.04175700
N	3.41396700	3.73422300	-1.27852300
N	-0.96387800	4.62406100	-1.64953300
N	-4.71086000	2.22795300	-1.89601300
N	-4.77097200	-2.13646500	-1.89400500
N	-5.37136600	0.05434100	-1.93534100

N	-3.09329700	3.82654900	-1.79518200
N	1.30823700	4.58382600	-1.47144700
N	5.01725200	2.12523300	-1.11133500
N	4.95216000	-2.22920700	-1.11424400
N	1.19247000	-4.61412900	-1.46974300
N	-3.19261600	-3.77338900	-1.79158700
N	1.21051200	-0.00491300	-0.05522800
C	0.77549800	1.23884600	-0.79203600
C	0.77055500	-1.23558600	-0.81085400
C	0.63214700	-0.01451600	1.32771200
C	2.71529500	-0.00851200	0.01867600
H	1.19896400	1.19272700	-1.79614300
H	-0.31059900	1.26619200	-0.83673000
H	1.15251700	2.11478300	-0.26683600
H	1.14167200	-2.12086100	-0.29710000
H	-0.31556300	-1.25626700	-0.85803200
H	1.19620600	-1.17682800	-1.81347400
H	0.97161600	-0.91043700	1.84578400
H	0.97480400	0.87201800	1.85963400
H	-0.45544700	-0.01197300	1.26616100
H	3.04179400	-0.91122700	0.52817400
H	3.08765700	0.00111500	-1.00102600
H	3.04497300	0.88240400	0.54655100
Cl	2.77106000	0.01759400	-3.53684800
C	-2.76242100	0.01082200	-0.64755300
O	-2.39829700	0.00682000	-1.75162100
O	-3.04531400	0.01426900	0.47607300

BP-TMACl-CO_2 (Orientation 2-near anion)

C	5.00755300	-1.52055400	4.10564200
C	5.07580800	-1.87332400	2.67820500
C	5.49416900	-0.90041000	1.76489100
C	5.61478400	0.41674100	2.21854800
C	5.52538200	0.67800500	3.66443100
C	5.50243700	1.49337000	1.33306700
C	5.09125800	2.72665300	1.84793500
C	5.02340400	2.89579200	3.30857500
C	4.45878200	3.67037300	1.03235000
C	3.61116300	4.60101100	1.64061600
C	3.60217100	4.69496300	3.10946000
C	3.57342500	-3.26584800	4.54547200
C	3.58164900	-3.69111000	3.13624600
C	4.43655200	-3.03834200	2.24267300
C	1.50353300	-4.20825900	4.89586300
C	1.42593000	-4.67363900	3.50154900
C	2.53685300	-4.49608700	2.67141100

C	-0.78929500	-4.16089400	5.08721700
C	-0.96247500	-4.62198500	3.70030100
C	0.17143600	-4.97927100	2.96471200
C	2.57122000	5.20063200	0.92295400
C	1.46344200	5.66591800	1.63760100
C	1.54068800	5.71776300	3.10645600
C	-2.85053600	-3.13698700	5.08356700
C	-3.10905200	-3.55480700	3.69623500
C	-2.18775500	-4.39586800	3.06525600
C	0.21045800	5.77719400	1.02612400
C	-0.92459000	5.71167600	1.83952000
C	-0.75194700	5.76190100	3.30027500
C	-2.15414200	5.29153500	1.32213600
C	-3.08098200	4.73148800	2.20639900
C	-2.82204400	4.82039000	3.65254200
C	-4.05515600	3.83721200	1.75150400
C	-4.57777600	2.91677100	2.66493700
C	-4.25859600	3.07740200	4.09266100
C	-5.11492000	1.70184300	2.22861400
C	-5.11629900	0.62705300	3.12307700
C	-4.77587800	0.87886100	4.53253600
C	-5.12049300	-0.69125500	2.65789900
C	-4.59133200	-1.68250700	3.49063900
C	-4.27333800	-1.33886200	4.88595400
C	-4.07598600	-2.86692300	2.95613700
H	5.50361200	-1.11653000	0.69932000
H	5.51336000	1.32379000	0.25920300
H	4.44671800	3.54373900	-0.04737400
H	4.42637000	-3.29603300	1.18634800
H	2.48800300	-4.78125000	1.62337500
H	0.07684900	-5.27293200	1.92224500
H	2.52315200	5.10421200	-0.15883500
H	-2.32777300	-4.67722700	2.02449700
H	0.11587700	5.69043200	-0.05362700
H	-2.29548900	5.19695100	0.24813400
H	-4.23347300	3.71384000	0.68586900
H	-5.31390300	1.53789200	1.17212300
H	-5.31868500	-0.90137100	1.60955200
H	-4.25317200	-3.11915000	1.91347900
N	2.62851300	-3.68040800	5.40529200
N	5.39559700	-0.31661900	4.55759800
N	4.42852500	3.96024200	3.87179500
N	0.45398300	5.93269200	3.86623800
N	-3.53681600	4.11578700	4.54538500
N	-4.53550900	-0.12328200	5.39403600
N	-1.79258300	-3.59104900	5.77521300

N	-3.55936700	-2.15999900	5.67335200
N	-4.52802000	2.11657100	4.99189100
N	-1.75855000	5.47772300	4.14325500
N	2.66228400	5.39164800	3.76935300
N	5.40372300	1.92320500	4.15322700
N	4.40594500	-2.31695000	5.00436700
N	0.41548000	-4.13449100	5.68008400
C	4.40264300	-2.60804300	-2.08195400
C	4.47928100	-2.96253100	-3.50496600
C	4.91508500	-1.99117800	-4.40723500
C	5.01968900	-0.67149200	-3.96325100
C	4.91987400	-0.41238600	-2.52095700
C	4.91528100	0.40812400	-4.84065900
C	4.48251400	1.63821400	-4.33738000
C	4.40769000	1.80236100	-2.87934500
C	3.85979800	2.59211000	-5.14498500
C	2.99373600	3.51436700	-4.54886800
C	2.97853500	3.59940400	-3.08179100
C	2.95312200	-4.34072100	-1.64963800
C	2.96036300	-4.76487400	-3.05605000
C	3.83825200	-4.12467800	-3.93461500
C	0.87242900	-5.26788300	-1.30360000
C	0.79080700	-5.73302300	-2.69502500
C	1.91213400	-5.56418900	-3.51348600
C	-1.42425400	-5.21654100	-1.11052900
C	-1.60444800	-5.68950800	-2.49052500
C	-0.46514600	-6.05329500	-3.21554300
C	1.96320200	4.13770500	-5.25863700
C	0.84210200	4.59075400	-4.55600600
C	0.91513800	4.62776900	-3.08818300
C	-3.49029100	-4.19345400	-1.11244400
C	-3.76196300	-4.62484500	-2.49189800
C	-2.84419800	-5.48405100	-3.10452300
C	-0.41203800	4.73686200	-5.15876200
C	-1.55540500	4.65171400	-4.35829400
C	-1.38106200	4.68507100	-2.89835100
C	-2.80106600	4.26392300	-4.86491200
C	-3.72720200	3.68061000	-3.99438900
C	-3.46096200	3.75687600	-2.54962200
C	-4.72832300	2.80721000	-4.43427900
C	-5.23884600	1.86514400	-3.53502500
C	-4.90918000	2.01985500	-2.10945600
C	-5.80439200	0.65546700	-3.95186200
C	-5.78434300	-0.43232000	-3.07201300
C	-5.43089900	-0.17938300	-1.66665400
C	-5.81222200	-1.75836300	-3.51454200

C	-5.25639100	-2.75008000	-2.69853500
C	-4.92186300	-2.39778200	-1.31065700
C	-4.75523500	-3.95029800	-3.21084900
H	4.95858200	-2.21406700	-5.46842200
H	4.96070200	0.24412300	-5.91266600
H	3.88284800	2.48753300	-6.22539000
H	3.85221900	-4.39767900	-4.98490000
H	1.87596000	-5.87082600	-4.55394300
H	-0.55787000	-6.37902900	-4.24654000
H	1.93847200	4.07986900	-6.34267100
H	-3.00328900	-5.81115200	-4.12760100
H	-0.50175900	4.70134800	-6.24038600
H	-2.95997000	4.21737800	-5.93813600
H	-4.94307500	2.71753700	-5.49494300
H	-6.04966400	0.50260100	-4.99852300
H	-6.05616000	-1.98069600	-4.54891300
H	-4.97221500	-4.23619900	-4.23572500
N	2.00293800	-4.75511900	-0.79397500
N	4.80299400	-1.40925300	-1.62959500
N	3.80787500	2.86817600	-2.32146200
N	-0.17462700	4.84731800	-2.33354800
N	-4.18406100	3.05668200	-1.66077600
N	-5.18886300	-1.18172700	-0.80753600
N	-2.42961500	-4.64565300	-0.42541600
N	-4.20205800	-3.21610800	-0.52712300
N	-5.18553300	1.06013900	-1.21249800
N	-2.39370400	4.40743200	-2.06079600
N	2.03480100	4.29696300	-2.42688400
N	4.80017800	0.83503600	-2.03625900
N	3.80137900	-3.40951800	-1.18687000
N	-0.21838000	-5.19275900	-0.52162200
N	0.28828900	-0.06547100	-0.20752600
C	-0.98004700	-0.09047800	-1.01955400
C	0.90517000	-1.44292900	-0.21799000
C	-0.01840300	0.34828000	1.19990600
C	1.25450100	0.91022300	-0.83460000
H	-0.72144700	-0.38977300	-2.03588700
H	-1.66957500	-0.80341400	-0.56853600
H	-1.42053000	0.90602000	-1.00714200
H	1.82502600	-1.41907100	0.36234800
H	0.19922000	-2.14971500	0.21534600
H	1.12460600	-1.69644500	-1.25537200
H	0.90643000	0.36733500	1.77384100
H	-0.46803300	1.33989300	1.18860200
H	-0.71068200	-0.36909800	1.63755000
H	2.18036100	0.90501300	-0.26339000

H	1.44457100	0.57413000	-1.85504700
H	0.80978100	1.90425100	-0.82277500
Cl	1.34464100	-1.18539800	-3.76841700
C	-1.34204800	-2.48073400	-4.87155500
O	-0.78499600	-3.36660400	-5.37515300
O	-1.99654400	-1.64470900	-4.39922000

BP-MIMCl-CO_2 (Orientation 1-near cation)

C	3.87569700	3.92047400	3.56410000
C	4.10321400	3.86038200	2.11091000
C	3.28088600	4.61548600	1.27202700
C	2.15294200	5.22253200	1.83170300
C	2.00692700	5.23367500	3.29648700
C	1.04127200	5.53284000	1.04492600
C	-0.20159600	5.63527400	1.67347900
C	-0.25441500	5.63273900	3.14439000
C	-1.38521700	5.43732500	0.95785900
C	-2.52374700	5.04725700	1.66477900
C	-2.48504100	5.06599900	3.13591400
C	4.96457700	1.92382000	3.89041100
C	5.24584400	1.78444500	2.45206500
C	4.89900300	2.83161200	1.59605000
C	5.02117700	-0.35124500	4.19996300
C	5.30383000	-0.58612500	2.77445300
C	5.53029300	0.51251600	1.94273000
C	4.03906500	-2.41618900	4.42635200
C	4.27218300	-2.73301500	3.00802600
C	5.01355700	-1.84172700	2.22955200
C	-3.56596700	4.36876100	1.02696900
C	-4.39873200	3.56414500	1.80594400
C	-4.27871400	3.63377500	3.27063000
C	2.25034900	-3.85743800	4.53356500
C	2.40509000	-4.22732000	3.11769200
C	3.49726000	-3.72820500	2.40448700
C	-5.09039400	2.49188000	1.23564300
C	-5.43379500	1.42268300	2.06572400
C	-5.26423400	1.57582400	3.51902900
C	-5.59216500	0.13763600	1.53821400
C	-5.35431400	-0.94866100	2.38500100
C	-5.19476900	-0.69877400	3.82705000
C	-4.95327800	-2.18638700	1.86952400
C	-4.22136100	-3.03479300	2.70314500
C	-4.10607600	-2.70020600	4.13099400
C	-3.34429500	-3.98113600	2.16418700
C	-2.27210700	-4.40196000	2.95289800
C	-2.23957100	-4.01802900	4.37235800

C	-1.10401800	-4.89323200	2.36408700
C	0.08293000	-4.81281300	3.09574900
C	0.01994300	-4.42146400	4.51271500
C	1.32083100	-4.80097100	2.44855900
H	3.38472800	4.54091700	0.19305900
H	1.10211600	5.47346600	-0.03799200
H	-1.36658100	5.36918000	-0.12598700
H	5.04253200	2.73182700	0.52330600
H	5.68921700	0.36746200	0.87676000
H	5.15480100	-2.02890000	1.16738800
H	-3.58972600	4.29027000	-0.05606700
H	3.60366900	-3.94164000	1.34321900
H	-5.14537600	2.38275500	0.15598000
H	-5.66818100	-0.01384700	0.46417800
H	-5.01081400	-2.38304400	0.80144200
H	-3.36450500	-4.20564200	1.10082600
H	-1.07907400	-5.12263500	1.30188900
H	1.38768500	-5.02720100	1.38718900
N	5.03829400	0.88131600	4.73380400
N	2.95239400	4.73104600	4.10708200
N	-1.41269600	5.50859300	3.81307600
N	-4.86471400	2.73150300	4.07367700
N	-4.73705800	-1.64176200	4.66624500
N	-1.14855600	-4.19588800	5.13495600
N	3.15897100	-3.10244500	5.17365900
N	1.12296000	-4.11589300	5.21631800
N	-3.23833500	-3.32472100	4.94279500
N	-5.33010500	0.52829600	4.35635600
N	-3.45033700	4.49924000	3.87778400
N	0.85949000	5.59538900	3.89404700
N	4.45539900	3.05309900	4.40972500
N	4.56642200	-1.32475100	5.00567400
C	4.08898000	2.92481700	-2.54529300
C	4.29473500	2.88351000	-3.99987000
C	3.46465500	3.66353100	-4.80884000
C	2.32245700	4.23780700	-4.24365500
C	2.18216400	4.19816800	-2.78121700
C	1.20565700	4.59610300	-5.00582600
C	-0.04458200	4.65382300	-4.38392500
C	-0.08727300	4.58312600	-2.91618000
C	-1.24700700	4.51549500	-5.08692500
C	-2.38229700	4.07237300	-4.40190600
C	-2.32370900	4.02496500	-2.93387600
C	5.24454000	0.95766900	-2.23803300
C	5.47430000	0.81574400	-3.68252300
C	5.10396300	1.86844500	-4.52019000

C	5.33677000	-1.31183100	-1.91175900
C	5.58341300	-1.55357700	-3.34045600
C	5.77827800	-0.45581700	-4.17744000
C	4.33353000	-3.35610400	-1.65658900
C	4.56798000	-3.70072400	-3.06645300
C	5.31714000	-2.82551200	-3.85055000
C	-3.45421900	3.43563000	-5.03839700
C	-4.27283800	2.58177300	-4.29149600
C	-4.14125600	2.60807600	-2.82688800
C	2.50406200	-4.73091200	-1.54488800
C	2.66682500	-5.14846800	-2.94450800
C	3.79221800	-4.70624400	-3.64081300
C	-4.99365900	1.52870100	-4.86643000
C	-5.32676300	0.42883900	-4.06734200
C	-5.17884400	0.56497200	-2.61020300
C	-5.50657500	-0.85684200	-4.58928600
C	-5.25435200	-1.95461500	-3.75703100
C	-5.12684700	-1.71176000	-2.31211400
C	-4.82756500	-3.19105800	-4.25163900
C	-4.04734500	-4.00776600	-3.42435900
C	-3.95481700	-3.66469900	-1.99785900
C	-3.13859900	-4.94080400	-3.93220800
C	-2.03886700	-5.30457700	-3.14930700
C	-2.01425100	-4.88485200	-1.74100500
C	-0.85902800	-5.81094600	-3.70196400
C	0.32973500	-5.69097200	-2.98104200
C	0.26003800	-5.24628500	-1.58216200
C	1.58344100	-5.73383200	-3.59587600
H	3.58781400	3.64845600	-5.88756800
H	1.27528800	4.62481200	-6.08905400
H	-1.24671800	4.54981600	-6.17220300
H	5.25521800	1.79001800	-5.59249800
H	5.94176600	-0.60262500	-5.24054100
H	5.47789200	-3.03686400	-4.90297700
H	-3.51580900	3.43400900	-6.12248600
H	3.92957100	-4.97471500	-4.68346300
H	-5.08675300	1.46248000	-5.94621600
H	-5.59863700	-0.99919700	-5.66178100
H	-4.89740400	-3.40523900	-5.31386600
H	-3.16950000	-5.21385600	-4.98277300
H	-0.83389600	-6.11748900	-4.74332200
H	1.67043400	-6.03587300	-4.63497900
N	5.38624500	-0.07140800	-1.39275900
N	3.14298200	3.70018400	-1.98863400
N	-1.24382400	4.43165800	-2.25148700
N	-4.75064900	1.70428800	-2.04450000

N	-4.65831500	-2.64517300	-1.47310100
N	-0.91924500	-5.00848700	-0.98112000
N	3.44542600	-4.02043100	-0.90570000
N	1.35920300	-4.94539100	-0.87796900
N	-3.05084200	-4.22879400	-1.18723900
N	-5.29256300	-0.48645500	-1.78559100
N	-3.28761000	3.43685300	-2.20697700
N	1.02919900	4.52588600	-2.17531300
N	4.72642400	2.08477100	-1.71730400
N	4.90849400	-2.27980700	-1.09191100
C	0.46567900	1.61971500	-0.44576100
C	-0.83831200	1.32897200	-0.70741300
C	0.25800200	-0.58271300	-0.46027800
H	0.97699600	2.56659000	-0.40020600
H	-1.68183300	1.97352800	-0.89646500
H	0.50009800	-1.63161700	-0.45761000
N	1.12447100	0.41340500	-0.27933200
N	-0.94837500	-0.05505800	-0.69490100
C	2.54596500	0.22280600	0.01659400
H	2.68915100	0.11884300	1.09305700
H	2.88680300	-0.66876300	-0.50524000
H	3.10043300	1.08262600	-0.34929200
C	-2.14902500	-0.82649000	-1.01355600
H	-2.12223500	-1.78325700	-0.49613500
Cl	1.83803900	-1.68244500	-3.01560900
H	-2.19163900	-1.00255700	-2.08951200
H	-3.03071100	-0.26937500	-0.70546700
C	-1.96626700	-0.07038700	2.42789200
O	-2.35387600	1.02227600	2.41032800
O	-1.57928800	-1.16560000	2.41645000

BP-MIMCl-CO_2 (Orientation 2-near anion)

C	4.78263700	2.57036900	3.80241300
C	4.97293200	2.51069000	2.34364500
C	4.39110500	3.50233500	1.54966300
C	3.48473700	4.37763700	2.15434300
C	3.36121100	4.36861900	3.62146700
C	2.49718200	5.01924600	1.40206100
C	1.33717300	5.43487300	2.05943700
C	1.30044900	5.38688400	3.53029300
C	0.13641700	5.59407100	1.36183500
C	-1.05725700	5.49012600	2.07822300
C	-0.99934500	5.43846000	3.54810900
C	5.26532500	0.33519300	4.03049300
C	5.48299800	0.18387700	2.58219100
C	5.44173800	1.32285800	1.77433200

C	4.69386200	-1.87951200	4.25386700
C	4.88022300	-2.12135100	2.81352700
C	5.39270800	-1.09327000	2.01905400
C	3.19894600	-3.61740700	4.43223400
C	3.31388800	-3.92356000	2.99688900
C	4.25155200	-3.22717700	2.23201100
C	-2.24626400	5.12701500	1.43842000
C	-3.24726500	4.52891400	2.20581800
C	-3.10024600	4.51065400	3.67026300
C	1.09733900	-4.54679800	4.53998800
C	1.12444300	-4.88780800	3.10846200
C	2.29415100	-4.65708200	2.38237000
C	-4.19731900	3.69059100	1.61318900
C	-4.80229900	2.72036200	2.41459400
C	-4.58928600	2.77090400	3.87014900
C	-5.32082400	1.55030900	1.84961500
C	-5.39604700	0.41440000	2.65905200
C	-5.15706600	0.55685600	4.10448500
C	-5.36931800	-0.86518700	2.09517000
C	-4.89786600	-1.91549300	2.88716000
C	-4.68072700	-1.68009000	4.32381200
C	-4.33506100	-3.05234600	2.30004100
C	-3.41845600	-3.79029400	3.05399900
C	-3.26416700	-3.48328000	4.48502600
C	-2.44422600	-4.56881500	2.42442300
C	-1.26968100	-4.84359700	3.13051200
C	-1.20277700	-4.50046200	4.56003600
C	-0.08475700	-5.13416500	2.45150000
H	4.46173400	3.44891400	0.46653900
H	2.52971700	4.99221900	0.31644700
H	0.12401400	5.56915300	0.27585100
H	5.54546000	1.23531000	0.69590500
H	5.49183400	-1.22762400	0.94472700
H	4.31745600	-3.39554200	1.16029300
H	-2.30267100	5.10134100	0.35377400
H	2.31755000	-4.84024200	1.31127900
H	-4.29453700	3.64351700	0.53154100
H	-5.44973200	1.46682700	0.77292800
H	-5.49598600	-0.99683900	1.02279800
H	-4.43390500	-3.22262400	1.23053400
H	-2.50143900	-4.75586300	1.35489500
H	-0.10146200	-5.32493500	1.38143000
N	5.05411000	-0.72167600	4.83167600
N	4.13243500	3.58617700	4.39366900
N	0.15895500	5.55516500	4.21810600
N	-3.89120200	3.75921300	4.45365400

N	-4.98220300	-0.50717600	4.90515100
N	-2.25662000	-3.98741200	5.21679400
N	2.18305300	-4.08062500	5.17919600
N	-0.04677500	-4.52704800	5.24407300
N	-4.02076200	-2.55750000	5.09771200
N	-4.93587500	1.75289500	4.67469700
N	-2.07063000	5.11141000	4.28952300
N	2.36785300	5.01384600	4.25503600
N	5.09886300	1.53911200	4.60232200
N	4.00603400	-2.72859600	5.03474900
C	4.68097200	1.92089500	-2.36441700
C	4.85092200	1.87852100	-3.82433800
C	4.25107800	2.88534300	-4.58808100
C	3.30259400	3.71155600	-3.97947700
C	3.17546900	3.65574000	-2.51526000
C	2.30953800	4.38069800	-4.70283500
C	1.12450500	4.73516500	-4.05158000
C	1.08187200	4.61970800	-2.58584900
C	-0.07970000	4.94724400	-4.73110900
C	-1.28380700	4.79146600	-4.03642100
C	-1.22284700	4.67137600	-2.57235800
C	5.22347700	-0.30123000	-2.14982400
C	5.39790800	-0.44843700	-3.60188000
C	5.34719000	0.70317100	-4.39444100
C	4.59026400	-2.49580700	-1.91487500
C	4.74694800	-2.74434400	-3.35494100
C	5.28782400	-1.72951900	-4.14919200
C	3.01303900	-4.16675300	-1.71401000
C	3.12355300	-4.50216900	-3.14075900
C	4.08297600	-3.84192900	-3.90864800
C	-2.49281600	4.48832600	-4.67090200
C	-3.50330700	3.85801600	-3.93540600
C	-3.35382900	3.79128500	-2.47442100
C	0.89747500	-5.06670500	-1.58480500
C	0.92861200	-5.45953300	-3.00032300
C	2.10289800	-5.25837800	-3.72156800
C	-4.48635900	3.06078700	-4.53031000
C	-5.10374900	2.06846400	-3.75981300
C	-4.90988000	2.10238400	-2.30299900
C	-5.63623400	0.90361500	-4.32155900
C	-5.70712400	-0.24717400	-3.52865600
C	-5.50764100	-0.10601800	-2.07924300
C	-5.65506400	-1.53389300	-4.07362300
C	-5.15512400	-2.57253600	-3.28350500
C	-4.96611700	-2.32874800	-1.84638500
C	-4.56387000	-3.71100800	-3.83873900

C	-3.62671700	-4.41390500	-3.08132000
C	-3.47832800	-4.07669500	-1.65868700
C	-2.64905700	-5.21504700	-3.67438900
C	-1.46604100	-5.44318100	-2.97469300
C	-1.39756400	-5.04527100	-1.56181100
C	-0.27832000	-5.75914400	-3.63223900
H	4.35464000	2.88143500	-5.66895200
H	2.37105900	4.43493100	-5.78556300
H	-0.08434400	5.02504400	-5.81411400
H	5.45854300	0.62583500	-5.47163200
H	5.38782600	-1.87434100	-5.22011500
H	4.15891000	-4.04278600	-4.97255300
H	-2.56561500	4.54515300	-5.75280400
H	2.14387700	-5.50814700	-4.77702600
H	-4.60474800	3.06122400	-5.60966700
H	-5.77014400	0.83097700	-5.39664900
H	-5.78350500	-1.67828200	-5.14208900
H	-4.67192500	-3.91904400	-4.89879400
H	-2.71727300	-5.46967600	-4.72726400
H	-0.29120600	-6.03100100	-4.68282300
N	5.02958400	-1.35730400	-1.34807700
N	3.98782000	2.89963500	-1.76149700
N	-0.06397200	4.74952400	-1.90072800
N	-4.17469100	3.05725800	-1.70998100
N	-5.33241000	-1.16560100	-1.27858800
N	-2.46631200	-4.54823100	-0.91927300
N	1.99185900	-4.59534100	-0.96216200
N	-0.24213200	-5.05275000	-0.88049700
N	-4.27909000	-3.17293200	-1.06611500
N	-5.30504000	1.09560200	-1.51135200
N	-2.29913200	4.33475800	-1.84565900
N	2.15554100	4.24585300	-1.87278700
N	5.06874400	0.90556100	-1.57848400
N	3.86532300	-3.30342600	-1.12928700
C	0.73221700	1.55679200	-0.24787500
C	-0.60152800	1.54882400	-0.52290400
C	0.06297700	-0.55181900	-0.31839500
H	1.42993800	2.37434400	-0.17770900
H	-1.28700000	2.36247900	-0.69910000
H	0.06803100	-1.62847500	-0.35789400
N	1.12157600	0.23559200	-0.11036100
N	-0.99943000	0.22274200	-0.55030400
C	2.46173200	-0.22937000	0.25005200
H	2.59659600	-0.15670100	1.33023200
H	2.58427500	-1.26272000	-0.06744500
H	3.20187200	0.38534900	-0.25574900

C	-2.33193500	-0.27251900	-0.90225400
H	-2.56237700	-1.15992500	-0.31643400
Cl	-0.40075000	-2.23528000	-3.10313200
H	-2.33102200	-0.54809000	-1.95812200
H	-3.06157700	0.50392100	-0.68792700
C	2.09313800	-0.56892000	-3.95938500
O	2.11633900	0.02706600	-2.96104900
O	2.18287100	-1.10177700	-4.98648300

BP-MPHP-CO_2 (Orientation 1-near cation)

C	0.39699400	5.30273400	3.94149500
C	0.74413200	5.36996600	2.51201900
C	-0.27129100	5.59360400	1.58260400
C	-1.59793200	5.53492000	2.02760300
C	-1.85101700	5.44808300	3.47609400
C	-2.64201800	5.24914500	1.14636200
C	-3.79995000	4.65892300	1.66656100
C	-3.94712200	4.57144900	3.12975400
C	-4.60905600	3.84795100	0.86646200
C	-5.32253200	2.81845200	1.48748000
C	-5.40712800	2.80486700	2.95802000
C	2.35941800	4.21073500	4.44210300
C	2.79616500	4.25008700	3.03684000
C	2.00976900	4.92811700	2.10604500
C	3.67617300	2.37498400	4.87379700
C	4.18237700	2.35163200	3.49171500
C	3.80362700	3.37258700	2.61852600
C	4.08938200	0.12917700	5.14017600
C	4.61403700	0.01373100	3.76969400
C	4.75607300	1.17343400	3.00253600
C	-5.63839900	1.64790800	0.79094900
C	-5.82593700	0.47465100	1.52554300
C	-5.92726400	0.56359000	2.99233600
C	3.50647600	-2.09361700	5.18469200
C	3.99413300	-2.29835200	3.81099800
C	4.65479600	-1.24619200	3.16683200
C	-5.63016600	-0.78031500	0.94028300
C	-5.35955500	-1.86403600	1.77826700
C	-5.49526400	-1.68575400	3.23423300
C	-4.71343500	-3.00629100	1.29401400
C	-4.04791600	-3.82340800	2.21073900
C	-4.21747300	-3.55454700	3.64888000
C	-3.00876700	-4.66538000	1.79994700
C	-2.03799200	-5.01472100	2.74266600
C	-2.27725600	-4.67890700	4.15667700
C	-0.73712500	-5.34661900	2.34416700

C	0.30345200	-5.10907900	3.24504500
C	-0.03273800	-4.78293200	4.64136500
C	1.60606900	-4.86031300	2.79245000
C	2.45544700	-4.12414500	3.62028900
C	2.03617000	-3.85080600	5.00499700
C	3.52503100	-3.39496100	3.08532200
H	-0.04152600	5.59089800	0.52268200
H	-2.46063800	5.25741600	0.07665000
H	-4.47758100	3.84822400	-0.21072300
H	2.28758800	4.92274100	1.05739800
H	4.13311700	3.35316900	1.58370600
H	5.11275100	1.11011500	1.97718400
H	-5.50393200	1.60049000	-0.28504900
H	4.99920300	-1.35981200	2.14110700
H	-5.47975700	-0.86894900	-0.13217800
H	-4.55204100	-3.13431400	0.22665400
H	-2.83459500	-4.84090000	0.74155000
H	-0.51728200	-5.55458400	1.30029900
H	1.87101400	-5.03211200	1.75196800
H	3.83132500	-3.54275800	2.05205200
N	2.92723700	3.38794600	5.33933200
N	-0.85637000	5.50688200	4.37643300
N	-4.86639900	3.78076000	3.70564100
N	-5.91728600	-0.52955900	3.77073500
N	-3.42606300	-4.12388900	4.57211400
N	0.88946300	-4.33398900	5.50808000
N	3.72060800	-0.94782000	5.85238100
N	2.68474600	-2.96967000	5.78416500
N	-1.30176400	-4.75014100	5.07702200
N	-5.05522100	-2.60949100	4.10284200
N	-5.86996400	1.74195500	3.63395400
N	-3.05983500	5.13255000	3.96674400
N	1.27271800	4.87560300	4.86614700
N	3.80178900	1.31872100	5.69384600
C	2.48639100	4.62278400	-1.83096500
C	2.82930600	4.71440500	-3.25699400
C	1.79450200	4.93058000	-4.16866600
C	0.47427400	4.81507300	-3.72643600
C	0.22642400	4.70241100	-2.28186000
C	-0.58353800	4.55576300	-4.59843000
C	-1.75239300	3.99223000	-4.08761300
C	-1.90084100	3.88702000	-2.62969700
C	-2.64720300	3.29478900	-4.89874500
C	-3.49915100	2.36279100	-4.30900700
C	-3.57033000	2.30921900	-2.84400000
C	4.45933100	3.54821300	-1.34655500

C	4.89219900	3.59527100	-2.75052100
C	4.10515100	4.30679800	-3.65848200
C	5.68450900	1.64700300	-0.94927700
C	6.18825500	1.61852000	-2.33120800
C	5.87310900	2.69105800	-3.16963800
C	5.91977700	-0.63046800	-0.73213900
C	6.44954300	-0.75857500	-2.09911300
C	6.69382800	0.41172100	-2.82421800
C	-4.02640000	1.29796400	-5.04050800
C	-4.36867400	0.13243300	-4.35632700
C	-4.42054400	0.17506900	-2.88973700
C	5.14101600	-2.79578700	-0.74349700
C	5.64379400	-3.02251600	-2.10775200
C	6.41279200	-2.01792600	-2.70425100
C	-4.28695300	-1.11187400	-4.98142400
C	-4.02236900	-2.23020500	-4.18895800
C	-4.13750400	-2.09370300	-2.73084100
C	-3.36502900	-3.34866200	-4.70028300
C	-2.65221500	-4.16323000	-3.81816600
C	-2.83399000	-3.95401700	-2.37543700
C	-1.58474500	-4.95366500	-4.24404300
C	-0.60554200	-5.31579000	-3.31681100
C	-0.85560500	-5.03684900	-1.89562300
C	0.68743300	-5.67398400	-3.70389300
C	1.73064200	-5.51541400	-2.78973200
C	1.39223900	-5.19543600	-1.39434000
C	3.06081400	-5.37863900	-3.19742000
C	3.95334400	-4.71198100	-2.35601000
C	3.52048500	-4.41368300	-0.98178900
C	5.10688500	-4.08510700	-2.83837200
H	2.00739900	4.99091800	-5.23160000
H	-0.43372600	4.61664400	-5.67178100
H	-2.54200700	3.32717000	-5.97816000
H	4.38223800	4.34443300	-4.70761800
H	6.20667400	2.69040800	-4.20291700
H	7.06464700	0.35278000	-3.84306000
H	-3.94375700	1.29013500	-6.12189700
H	6.78498400	-2.14687100	-3.71613900
H	-4.18876200	-1.17282600	-6.06018300
H	-3.23094000	-3.45896900	-5.77137000
H	-1.41362000	-5.11024700	-5.30424800
H	0.90500100	-5.87859900	-4.74770000
H	3.33958600	-5.59805700	-4.22364200
H	5.44453600	-4.27474200	-3.85281300
N	5.00767300	2.69916200	-0.46595900
N	1.22691200	4.77962100	-1.39173100

N	-2.89613500	3.17608100	-2.07090000
N	-4.52037100	-0.94423000	-2.15644800
N	-2.02487700	-4.53172000	-1.47129000
N	2.32800400	-4.81792300	-0.51228700
N	5.45295900	-1.68970500	-0.05346500
N	4.23108800	-3.60738000	-0.18123500
N	0.11636400	-5.13464900	-0.97462900
N	-3.70584600	-3.05404600	-1.89712700
N	-4.19658900	1.30784000	-2.20471700
N	-0.98487500	4.39167200	-1.79031700
N	3.38003700	4.21957300	-0.91605200
N	5.73591900	0.56648800	-0.15644700
C	-1.59135100	-0.10935100	-0.25157300
C	-1.25134400	-1.43963900	-0.40390500
C	-0.98596900	-2.21132000	0.71846700
C	-1.10127800	-1.63418900	1.98493800
C	-1.44051500	-0.30150000	2.08972300
N	-1.66532700	0.44012300	0.97758000
H	-0.69999600	-3.24702600	0.59342100
H	-1.79828500	0.53696600	-1.08913300
H	-1.18148300	-1.83749200	-1.40104200
H	-0.92883700	-2.20554000	2.88753700
H	-1.53587300	0.20945900	3.03831600
C	-1.89354200	1.89740000	1.09841000
H	-2.48808900	2.09217600	1.98792700
H	-0.92587500	2.39403400	1.16705600
H	-2.41142700	2.25013400	0.20920300
P	-0.39256600	-0.19568000	-4.10393900
F	-0.28832000	1.37305400	-3.60034600
F	-1.30896600	0.27771500	-5.38027800
F	-0.55662200	-1.76248200	-4.55666500
F	0.95657900	-0.03553600	-4.97840800
F	0.47716200	-0.66388900	-2.79299900
F	-1.77491400	-0.34752100	-3.19966100
C	1.71115900	1.42139800	-1.35032000
O	0.93665900	1.38046400	-0.48789600
O	2.50442200	1.47738100	-2.19476300

BP-MPHP-CO_2 (Orientation 2-near anion)

C	5.68151400	0.89416700	3.61293400
C	5.75993700	0.73455000	2.15110400
C	5.52920700	1.84692200	1.33894300
C	5.01795400	3.00093600	1.94164400
C	4.97624300	3.07275400	3.41214900
C	4.27340500	3.92249200	1.20219900
C	3.35545400	4.72365700	1.88881100

C	3.38847700	4.73477900	3.36128900
C	2.23792900	5.25219600	1.23876100
C	1.11668300	5.56731100	2.01179000
C	1.24373800	5.55597500	3.47935400
C	5.35656600	-1.36106600	3.91691300
C	5.42106500	-1.61369200	2.46777500
C	5.74015100	-0.55584900	1.61325000
C	4.07042400	-3.23529300	4.26244700
C	4.07244500	-3.55887800	2.82617200
C	4.85759700	-2.78919900	1.96414200
C	2.09053600	-4.35874800	4.58896900
C	2.00268900	-4.71502800	3.16307700
C	3.07266600	-4.39501600	2.32337300
C	-0.16237400	5.59460400	1.44980600
C	-1.25678500	5.39488700	2.29510200
C	-1.03424200	5.38998100	3.75135200
C	-0.18937800	-4.52061500	4.84595600
C	-0.37266600	-4.87554500	3.42853100
C	0.76009900	-5.07217700	2.63439200
C	-2.47245000	4.91281300	1.80111000
C	-3.32268900	4.24588600	2.68580400
C	-3.00910500	4.27210700	4.12452800
C	-4.26044800	3.31944900	2.21952000
C	-4.67659000	2.31364000	3.09423000
C	-4.29389900	2.40600800	4.51312000
C	-5.15204800	1.09357900	2.60392500
C	-5.01860100	-0.03282500	3.41874200
C	-4.61980000	0.15269000	4.82385500
C	-4.93562400	-1.31081400	2.85863300
C	-4.27044700	-2.30025900	3.58699700
C	-3.91498800	-2.02813600	4.98994600
C	-3.66820800	-3.38236900	2.93935200
C	-2.60536600	-4.02140900	3.58274400
C	-2.32995700	-3.69303100	4.99156700
C	-1.63484400	-4.71227000	2.85242400
H	5.52947800	1.74889600	0.25870200
H	4.24284600	3.86167200	0.11968100
H	2.16195600	5.21064800	0.15781400
H	5.74720000	-0.70033500	0.53840300
H	4.84265900	-2.97483600	0.89596100
H	3.01579900	-4.60053100	1.26044000
H	-0.28937700	5.55109000	0.37383700
H	0.65744300	-5.28382600	1.57601100
H	-2.64943900	4.86039100	0.73276100
H	-4.48162900	3.24812000	1.16067700
H	-5.40128600	0.98338500	1.55451200

H	-5.17562900	-1.46550200	1.81243800
H	-3.86968700	-3.57456400	1.89124500
H	-1.78680000	-4.91736700	1.79851600
N	4.83970100	-2.25923700	4.77158600
N	5.46493100	2.08945400	4.18534200
N	2.41355500	5.30327100	4.08837000
N	-1.97990600	4.98305800	4.61289200
N	-4.44357700	1.37243000	5.35714800
N	-3.09686700	-2.83565800	5.68410900
N	1.02822200	-4.43913700	5.40664200
N	-1.21090400	-4.10258100	5.61067500
N	-4.25374100	-0.88060200	5.59940600
N	-3.62860100	3.46553400	5.00102600
N	0.17307000	5.63640300	4.28502400
N	4.30427400	4.04060200	4.05608100
N	5.65629800	-0.16205300	4.44227200
N	3.18571400	-3.78326900	5.11160800
C	5.05470000	1.19657400	-2.59154000
C	5.11726300	1.08498200	-4.05649500
C	4.60050900	2.13629200	-4.81818000
C	3.81335200	3.09799800	-4.17568500
C	3.81513900	3.12452400	-2.70575200
C	2.82813100	3.82834100	-4.84402800
C	1.75120400	4.33303300	-4.10663700
C	1.83940400	4.29914100	-2.64044900
C	0.51220300	4.62051200	-4.68208000
C	-0.62487900	4.63806400	-3.86875000
C	-0.44184400	4.56760200	-2.41303600
C	5.23668100	-1.07505800	-2.32649200
C	5.31208300	-1.29258800	-3.77973900
C	5.39510200	-0.16942600	-4.60918300
C	4.32430700	-3.15579100	-1.97694900
C	4.36680500	-3.47212400	-3.41261500
C	4.99863100	-2.56472300	-4.26979700
C	2.55413000	-4.57643100	-1.62655800
C	2.52123200	-4.96376800	-3.04411300
C	3.53345500	-4.48997000	-3.88387800
C	-1.91295100	4.46568600	-4.37813200
C	-2.92953700	4.04287400	-3.52102800
C	-2.65305900	3.99189100	-2.08030000
C	0.33143000	-5.10194500	-1.34164200
C	0.19910800	-5.50751500	-2.74807800
C	1.34969500	-5.53827500	-3.54034200
C	-4.08402200	3.41704900	-3.99355300
C	-4.80763900	2.60210600	-3.12384000
C	-4.46696800	2.62800000	-1.69610900

C	-5.60546200	1.55770300	-3.59451000
C	-5.82283100	0.46636900	-2.75355000
C	-5.44939500	0.59036800	-1.33835100
C	-6.01040500	-0.81229100	-3.27908100
C	-5.57400800	-1.90008200	-2.52197600
C	-5.21950200	-1.67349600	-1.11538900
C	-5.14989700	-3.08071900	-3.13158600
C	-4.21841900	-3.87671800	-2.46374400
C	-3.91717600	-3.56713800	-1.06074400
C	-3.37221700	-4.75073200	-3.14539400
C	-2.16563000	-5.11911500	-2.54850300
C	-1.93950000	-4.74271800	-1.14659000
C	-1.08462400	-5.60065300	-3.28853400
H	4.60975500	2.07853000	-5.90231600
H	2.78708600	3.82214800	-5.92895700
H	0.40533800	4.64207300	-5.76230900
H	5.43239000	-0.29198100	-5.68736200
H	5.02985900	-2.75857100	-5.33778500
H	3.52487800	-4.74147000	-4.94007300
H	-2.07971900	4.48045800	-5.45048700
H	1.28058300	-5.81777600	-4.58719900
H	-4.29429500	3.39823700	-5.05792100
H	-5.83799800	1.48520800	-4.65196200
H	-6.24455300	-0.94034900	-4.33051000
H	-5.36504600	-3.25875100	-4.17992800
H	-3.55627900	-4.98261200	-4.18961000
H	-1.21788000	-5.87211500	-4.33123700
N	4.92425300	-2.06357800	-1.47660200
N	4.57887600	2.29400200	-1.98294500
N	0.78010200	4.55641000	-1.85747800
N	-3.50957600	3.43485500	-1.21222300
N	-5.37367900	-0.47625600	-0.52890100
N	-2.88316900	-4.12775700	-0.41753600
N	1.52352800	-4.80943200	-0.80139100
N	-0.73948400	-4.88125700	-0.56359900
N	-4.56670700	-2.59843400	-0.39541500
N	-4.97777200	1.73993800	-0.83031200
N	-1.47185500	4.38350700	-1.57431600
N	2.93930500	3.86491800	-2.00896800
N	5.30707000	0.15301800	-1.78968700
N	3.55576900	-3.84135900	-1.11992300
C	-0.88567800	1.33719500	0.17059600
C	-2.00059100	0.53736400	0.33221400
C	-1.84515200	-0.84266500	0.41841800
C	-0.56578900	-1.39363300	0.34559600
C	0.51762000	-0.55193300	0.20231200

N	0.34976900	0.78985400	0.12270900
H	-2.71299900	-1.48540000	0.49966000
H	-0.95212900	2.41013400	0.03831300
H	-2.98058600	0.99560500	0.32736000
H	-0.41258900	-2.46589200	0.36727900
H	1.53579600	-0.91355200	0.13773100
C	1.53774600	1.66446500	-0.02381900
H	2.03440800	1.76147700	0.94206300
H	2.22503200	1.23214100	-0.74763900
H	1.22322400	2.64278600	-0.37731600
P	-1.91041300	-0.39785500	-3.49353400
F	-2.01188600	1.08547900	-4.18467500
F	-3.14761100	-0.92870800	-4.42899400
F	-1.82692000	-1.86893900	-2.76608300
F	-0.82478900	-0.87139100	-4.61058300
F	-0.68171000	0.14259600	-2.53469500
F	-2.98972900	0.08250000	-2.34983900
C	1.76356400	-0.89325700	-3.07210200
O	2.15099200	0.19014800	-3.23461800
O	1.43631600	-1.99105900	-2.89728400

1. Kresse, G. and J. Furthmüller, *Efficient iterative schemes for ab initio total-energy calculations using a plane-wave basis set.* Physical review B, 1996. **54**(16): p. 11169.
2. Janesko, B.G., T.M. Henderson, and G.E. Scuseria, *Screened hybrid density functionals for solid-state chemistry and physics.* Physical Chemistry Chemical Physics, 2009. **11**(3): p. 443-454.
3. Ramzan, M., et al., *Electronic, mechanical and optical properties of Y2O3 with hybrid density functional (HSE06).* Computational materials science, 2013. **71**: p. 19-24.
